# Silver Nanoparticles: Technological Advances, Societal Impacts, and Metrological Challenges

**DOI:** 10.3389/fchem.2017.00006

**Published:** 2017-02-21

**Authors:** Bryan Calderón-Jiménez, Monique E. Johnson, Antonio R. Montoro Bustos, Karen E. Murphy, Michael R. Winchester, José R. Vega Baudrit

**Affiliations:** ^1^Material Measurement Laboratory, Chemical Sciences Division, National Institute of Standards and TechnologyGaithersburg, MD, USA; ^2^Chemical Metrology Division, National Laboratory of MetrologySan Jose, Costa Rica; ^3^National Laboratory of Nanotechnology, National Center of High TechnologySan Jose, Costa Rica

**Keywords:** silver nanoparticles, synthesis, characterization, environment health and safety, metrology, reference materials

## Abstract

Silver nanoparticles (AgNPs) show different physical and chemical properties compared to their macroscale analogs. This is primarily due to their small size and, consequently, the exceptional surface area of these materials. Presently, advances in the synthesis, stabilization, and production of AgNPs have fostered a new generation of commercial products and intensified scientific investigation within the nanotechnology field. The use of AgNPs in commercial products is increasing and impacts on the environment and human health are largely unknown. This article discusses advances in AgNP production and presents an overview of the commercial, societal, and environmental impacts of this emerging nanoparticle (NP), and nanomaterials in general. Finally, we examine the challenges associated with AgNP characterization, discuss the importance of the development of NP reference materials (RMs) and explore their role as a metrological mechanism to improve the quality and comparability of NP measurements.

## Defining nanomaterials and nanoparticles: their importance in nanoscience, and nanotechnology

**Figure 1:**
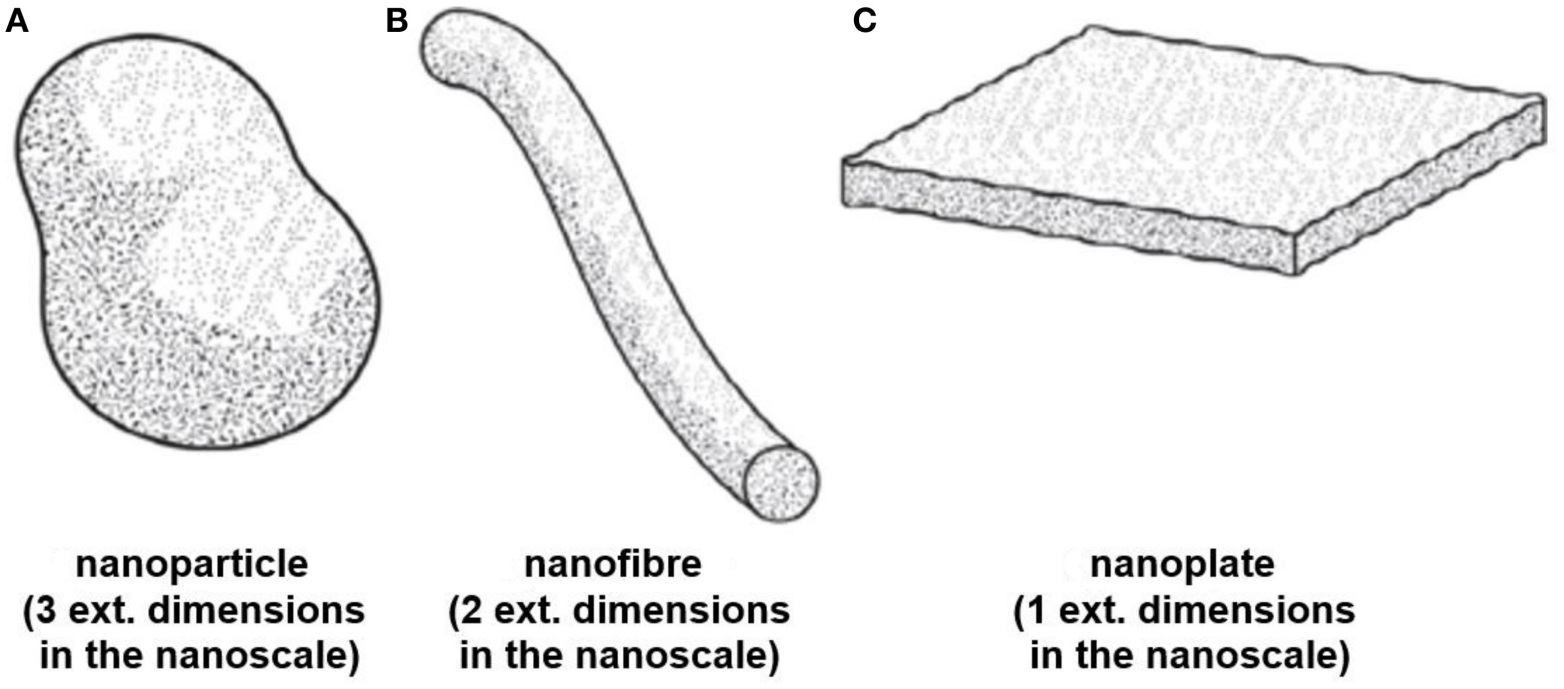
**Schematic diagrams displaying shape designations for nano-objects (A)** Nanoparticle, **(B)** Nanofiber **(C)** Nanoplate ©ISO. This material is excerpted from ISO 80004-2:20015 with permission from the American National Standard Institute (ANSI) on behalf of ISO. All rights reserved.

Standardization of vocabulary and nomenclature used in nanotechnology and nanoscience creates a common language through which research and industrial activities can be defined. Moreover, robust and well-founded definitions of the terms in these fields are essential to the formation of legally defensible and beneficial regulations to protect the environment and human health (ISO/TS 80004-1, 2015). Currently, an internationally harmonized definition for the term “nanomaterial” has not been established (Lövestam et al., 2010). Rather, a wide range of definitions are being used by different national authorities, scientific committees, and international organizations (Lidén, 2011; Boverhof et al., 2015; Contado, 2015), a few of which are discussed in this manuscript. The International Organization for Standardization (ISO) develops voluntary, consensus-based standards through the participation of over 160 national standards bodies and has been active in the promotion of uniform terminology in the field of nanotechnology. ISO defines a nanomaterial “as a material having any external dimension in the nanoscale or having internal structure or surface structure in the nanoscale” (ISO/TS 80004-1, 2015). The term “nanoscale” is further defined by ISO as the “length range approximately from 1 to 100 nm” (ISO/TS 80004-1, 2015). ISO classifies nanomaterials in two main categories: Nano-objects and nanostructured materials. A nano-object is described as a “discrete piece of material with one, two or three external dimensions in the nanoscale” (ISO/TS 80004-1, 2015) and a nanostructured material is a “material having internal structure or surface structure in the nanoscale” (ISO/TS 80004-4, 2011). Nano-objects, can be classified into three categories (see Figure 1) depending on their size and shape characteristics (ISO/TS 80004-1, 2015):

1. Nanoparticle (NP): “Nano-object with all external dimensions at the nanoscale where the lengths of the longest and shortest axes of the nano-object do not differ significantly”,
2. Nanofiber: “Nano-objects with two external dimensions at the nanoscale and the third dimension significantly larger”,
3. Nanoplate: “Nano-objects with one external dimension in the nanoscale and the other two dimensions significantly larger”,

ISO also provides a simple and general definition for engineered nanomaterials indicating that they are “nanomaterials designed for specific purposes or functions” (ISO/TS 80004-1, 2015).

Other organizations, federal agencies, and government bodies have developed their own approach to categorizing nanomaterials with the goal of assessing and controlling risk. The United States Environmental Protection Agency (U.S. EPA) has developed reporting and recordkeeping requirements for companies that manufacture or process nanoscale chemical substances. The entity describes “nanoscale chemical substances” as “chemical substances containing primary particles, aggregates, or agglomerates in the size range of 1 to 100 nm in at least one dimension” (EPA, 2015). The U.S. Food and Drug Administration (U.S. FDA) has issued a series of guidance documents with respect to the use of nanotechnology in FDA-regulated products (Hamburg, 2012; U.S. FDA, 2015). For example, when considering whether an U.S. FDA-regulated product involves nanotechnology, the U.S. FDA offers “Points to Consider” such as whether a material or product is engineered to have at least one dimension in the nanoscale range, or whether it exhibits chemical or physical properties or biological effects attributable to its dimensions (Croce, 2014; U.S. FDA, 2014). In recent years, the European Union (EU) has been engaged in a number of efforts to define “nanomaterials” and “engineered nanomaterial.” Particularly, the European Commission recommended the following definition for a nanomaterial: “Nanomaterial means a natural, incidental, or manufactured material containing particles, in an unbound state or as an aggregate or as agglomerate and where, for 50% or more of the particles in the number size distribution, one or more external dimensions in the size range 1–100 nm” (Commission Recommendation, 2011). The definitions cited in this directive are generally based on the ISO definition; however, they have been adapted with the goal of incorporating other technical concepts such as aggregation/agglomeration, particle size distribution, and particle number concentration (Commission Recommendation, 2011). Additionally, the EU has issued a series of directives in the fields of cosmetics (Regulation (EC) No 1223/2009), biocides (Regulation (EU) No 528/2012), food (Regulation (EU) No 1363/2013, Regulation (EU) No 1363/2013), and any food that was not used for human consumption to a significant degree, commonly denominate “novel food”. (Regulation (EU) 2015/2283). Recently, extensive technical work has begun to focus on the goal of providing recommendations on the possible use and limitations of some measurement techniques (MTs) with respect to the application of the EU definition (Babick et al., 2016).

Efforts to adapt and/or recast existing regulations to define fundamental concepts and applications of nanomaterials in consumer products are taking place in France (Decree No 2012-232) Belgium (Decree No 2014/24329), Denmark (Decree No 644 of 13/06/2014), and Canada (Health Canada, 2011). These countries have recently enacted their own policies to study the potential risks associated with the commercialization of nanomaterials by collecting information and establishing inventories. For instance, with the goal of identifying and assessing potential risks and benefits, Canadian regulatory agencies request information from manufacturers and other stakeholders on physical-chemical properties such as composition, purity, morphology, particle size/size distribution, chemical reactivity, agglomeration/aggregation state, as well as information on the methods used to assign these properties (Health Canada, 2011).

Despite efforts in recent years to properly define nanotechnology-related terms, more work needs to be done with respect to the harmonization and standardization of the terminology used in this field. For example, the term “nanoparticle” is defined differently by ISO (ISO/TS 80004-2, 2015), ASTM (ASTM E2456-06, 2012), and IUPAC (Alemán et al., 2007) with regard to the number of dimensions and shapes that can be attributed to NPs. This however, does not imply that one definition is accurate while another is not; rather it demonstrates that definitions and terms in the nanotechnology field are still evolving and highlights the importance of generating robust descriptors for these emerging materials to satisfy the variety of angles where the terminology would be applied.

## Impacts of the nanoparticles and silver nanoparticles (AgNPs) on commerce, technology and society

**Figure 2:**
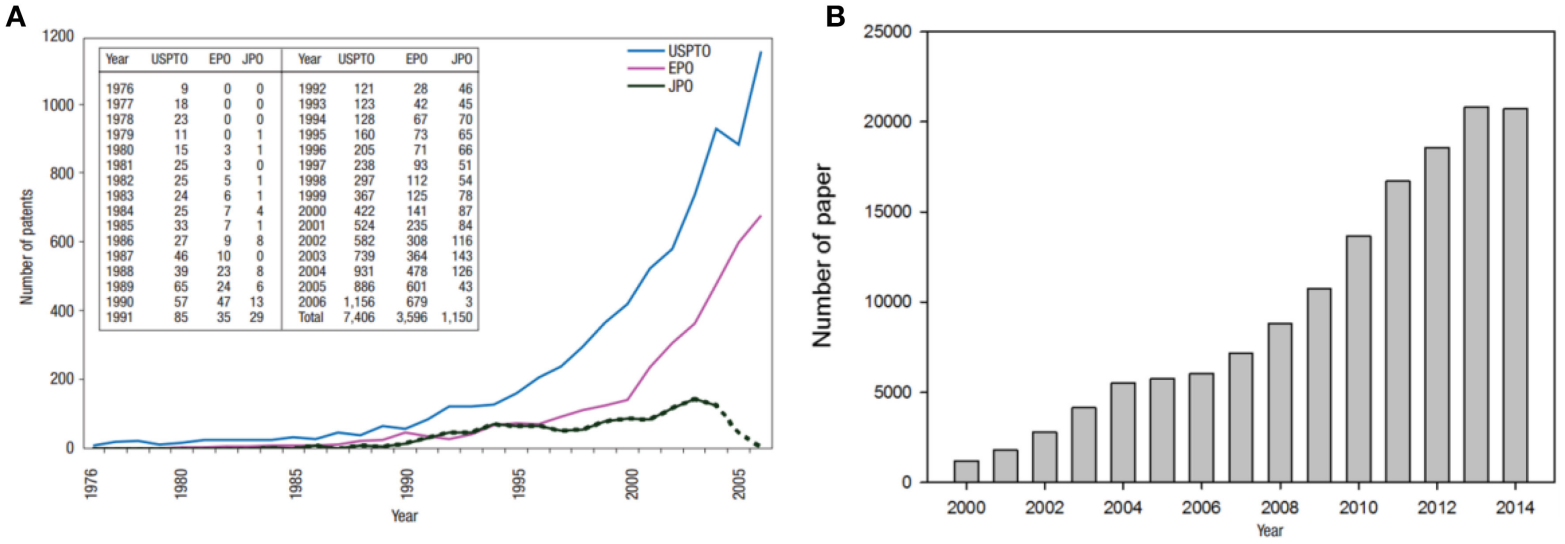
**(A)** Number of nanotechnology patents published by the United State Patent and Trademark Office (USPTO), the European Patent Office (EPO), and the Japan Patent Office (JPO) between 1976 and 2005, demonstrating the exponential growth of this emerging technology. The drop in the number of USPTO patents in 2005 is due to the USPTO enforcing a stricter definition of the term “nanotechnology.” The decline in the number of JPO patents for 2005 and 2006 is due to the delay between the publication and granting of patents at the JPO (Chen et al., 2008a). Reprinted by permission from Macmillan Publishers Ltd: [Nature Nanotechnology], copyright (2008) **(B)** Number of nanoscience papers indexed in Scopus® Elsevier between 2000 and 2014 by Shin et al. (2015) CC BY 2.0. This figure demonstrates the quick and substantial advances in the investigation in the nanoscience field.

In the past decade, the world has seen an exponential growth in the application of nanoscience and nanotechnology, leading to great strides in the development of new nanomaterials (see Figure 2). (López-Lorente and Valcárcel, 2016). This increase in innovation is largely due to the special properties that these materials possess at the nanoscale, leading to enhancement of mechanical (Calahorra et al., 2016), dimensional (Lee et al., 2011), electrical (Segev-Bar and Haick, 2013), magnetic (Reddy et al., 2012), photochemical (Watanabe et al., 2006), and catalytic (Gawande et al., 2016) attributes, to name a few. In general terms, NP applications are impacting different fields such as biomaterials (Ediriwickrema and Saltzman, 2015), composites (Ahmad et al., 2015), ceramics (Birol et al., 2013), polymers (Pecher and Mecking, 2010), food (Tiede et al., 2008), agriculture (Parisi et al., 2015; Phogat et al., 2016), and energy (Lohse and Murphy, 2012).

**Figure 3:**
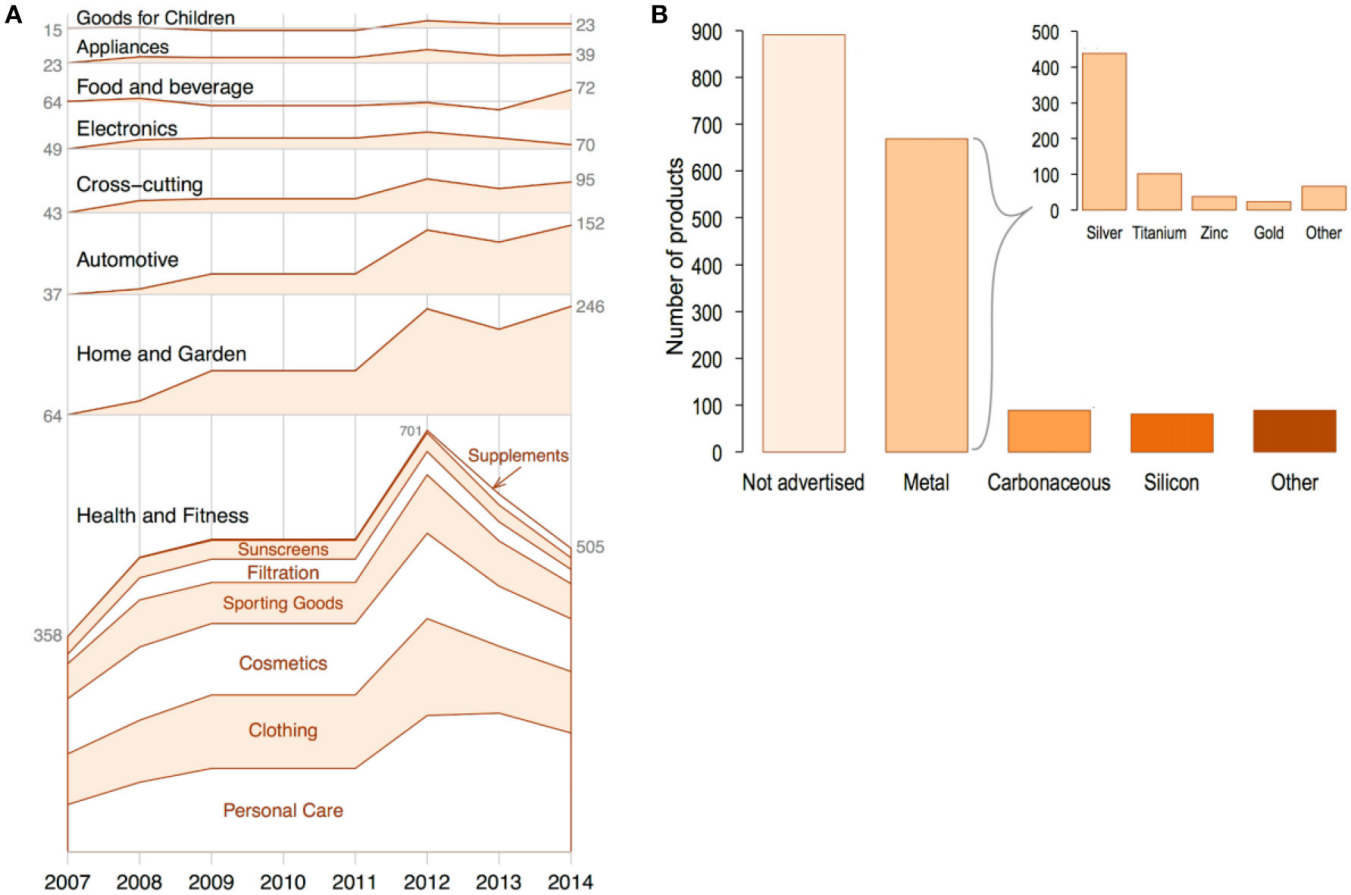
**(A)** Number of available nanomaterial-containing consumer products over time (since 2007) by category (black print) and sub category (red print). **(B)** Claimed composition of nanomaterials listed in the Nanotechnology Consumer Product Inventory, grouped into five major categories: Not advertised, metal (including metals and metal oxides), carbonaceous nanomaterials (carbon black, carbon nanotubes, fullerenes, graphene), silicon-based nanomaterials (silicon and silica), and other (organics, polymers, ceramics, etc.). The insert in 3b shows the claimed elemental composition of nanomaterials listed in the metals category: silver, titanium, zinc, gold, and other metals (magnesium, aluminum oxide, copper, platinum, iron, and iron oxides, etc.). Adapted from Vance et al. (2015) with the permission of Beilstein-Institut. CC BY 2.0.

All of this escalation in the research and development of new NP applications will have a direct impact on commerce and society. In 2011, it was estimated that US$ 65 billion had been invested into the nanotechnology field (Miller and Wickson, 2015). Moreover, it was projected that a cumulative investment of US$150 billion would be made by the private sector into the field by 2015 (Cientifica, 2011). It was further predicted that nanotechnology in the form of NPs would impact different fields such as electronics, information technology and manufactured goods in health care and life sciences (Lux Research, 2008; Fiorino, 2010; Sargent, 2016). These projections are reflected in the growth of the numbers of consumer products incorporating NPs into their formulations. These numbers have grown from a total of 54 products identified in 2005 to over 1,800 nanomaterial- and NP-containing consumer products in 2014 produced by 622 companies in 32 countries (Vance et al., 2015). The variety of products ranged from goods for children to personal care products (Figure 3), with metals and metal oxides being the most commonly used NPs in commercial products. Although, silicon dioxide NPs (SiO_2_-NPs), titanium oxide NPs (TiO_2_-NPs), and zinc oxide NPs (ZnO-NPs), are produced in the greatest quantities worldwide, with a global production of 5,500 t per year, 3,000 t per year and 550 t per year, respectively (Piccinno et al., 2012; Keller et al., 2013).

In recent years, there have been various estimates of the global production of AgNPs. (Whiteley et al., 2013). Mueller and Nowack (2008), estimated a worldwide AgNP production of 500 t per year for 2009, while Gottschalk et al. (2009) estimated 320 t for this same year. In the U.S. alone, Hendren et al. estimated in 2011 that between 2.8 t and 20 t of AgNPs would potentially be produced per year Hendren et al. (2011). It is projected that the global nanotechnology industry will continue to grow significantly. Specifically, the production of AgNPs is expected to reach approximately 800 t by 2025 (Pulit-Prociak and Banach, 2016). Vance et al. (2015) showed that AgNPs have greater marketing value than other NPs and their presence in consumer products are more widely advertised. This noted popularity can be attributed to the well-documented antimicrobial properties of ionic silver (Le Ouay and Stellacci, 2015). It should be clear that AgNPs by themselves have no antibacterial or antifungal properties, but it is the release of silver ions due to the destabilization of the AgNPs which confers such properties. Other distinctive physico-chemical properties of AgNPs such as high electrical and thermal conductivity (Alshehri et al., 2012), surface-enhanced Raman scattering (Nie and Emory, 1997), catalytic activity (Xu et al., 2006), and non-linear optical properties (Kelly et al., 2003), have led to a variety of new products and scientific applications (Tran et al., 2013).

**Figure 4:**
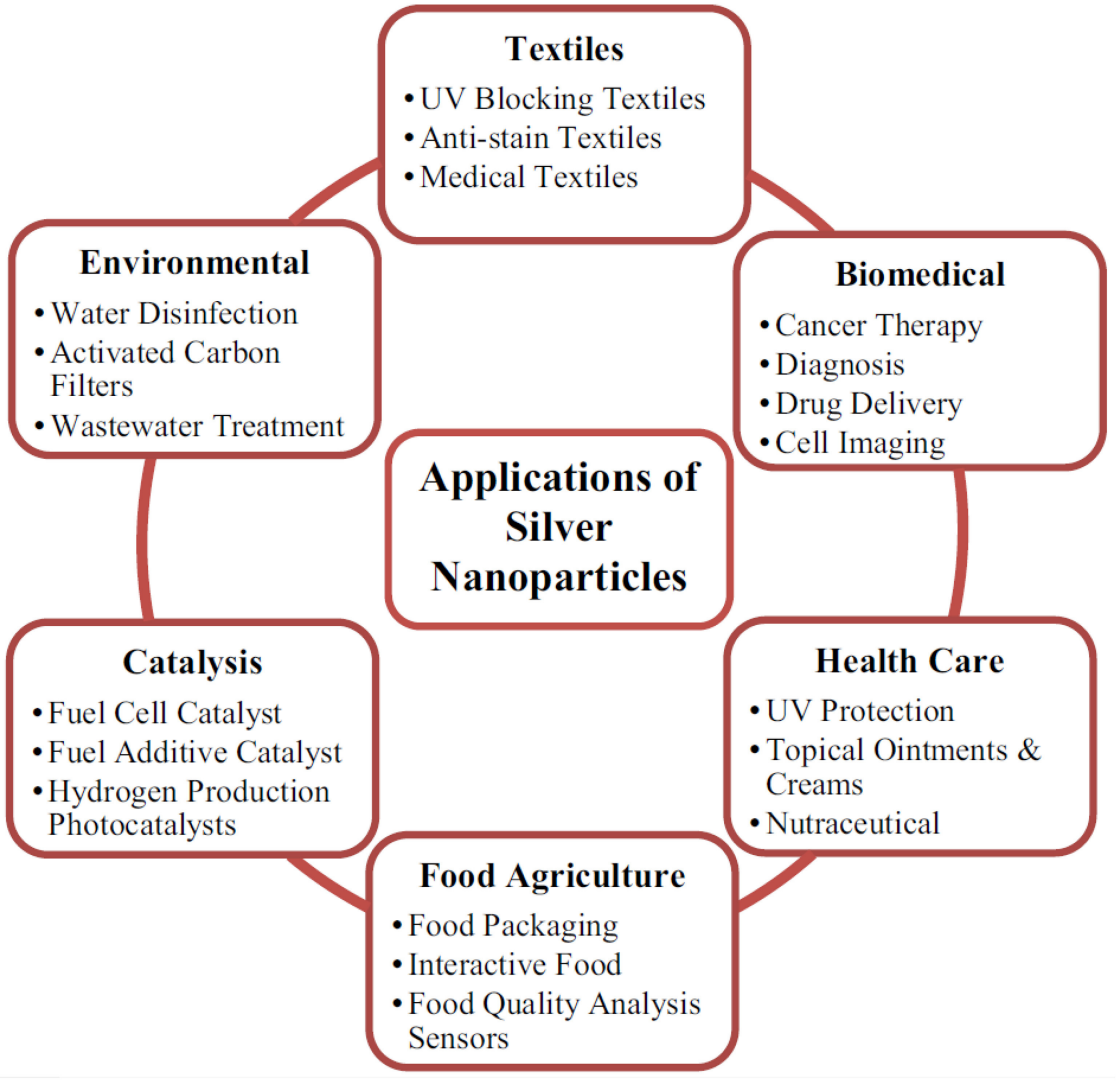
**Applications of AgNPs**. Reproduced from Keat et al. (2015) with permission of Bioresources and Bioprocessing. CC BY 4.0

The physico-chemical properties mentioned above offer AgNPs the capability of being used in a plethora of new commercial and technological applications, including as antiseptic agents in the medical field, cosmetic, food packaging, bioengineering, electrochemistry, and catalysis industries (Keat et al., 2015). As displayed in Figure 4, the antibacterial and antimicrobial activity of AgNPs are among the main reasons for their use in the formulation of surface cleaners, toys, textiles, air and water disinfection, antimicrobial catheters, antimicrobial gels, antimicrobial paints, food packaging supplies, clinical clothing, and food preservation etc. (Wijnhoven et al., 2009; Tolaymat et al., 2010; Tran et al., 2013).

**Figure 5:**
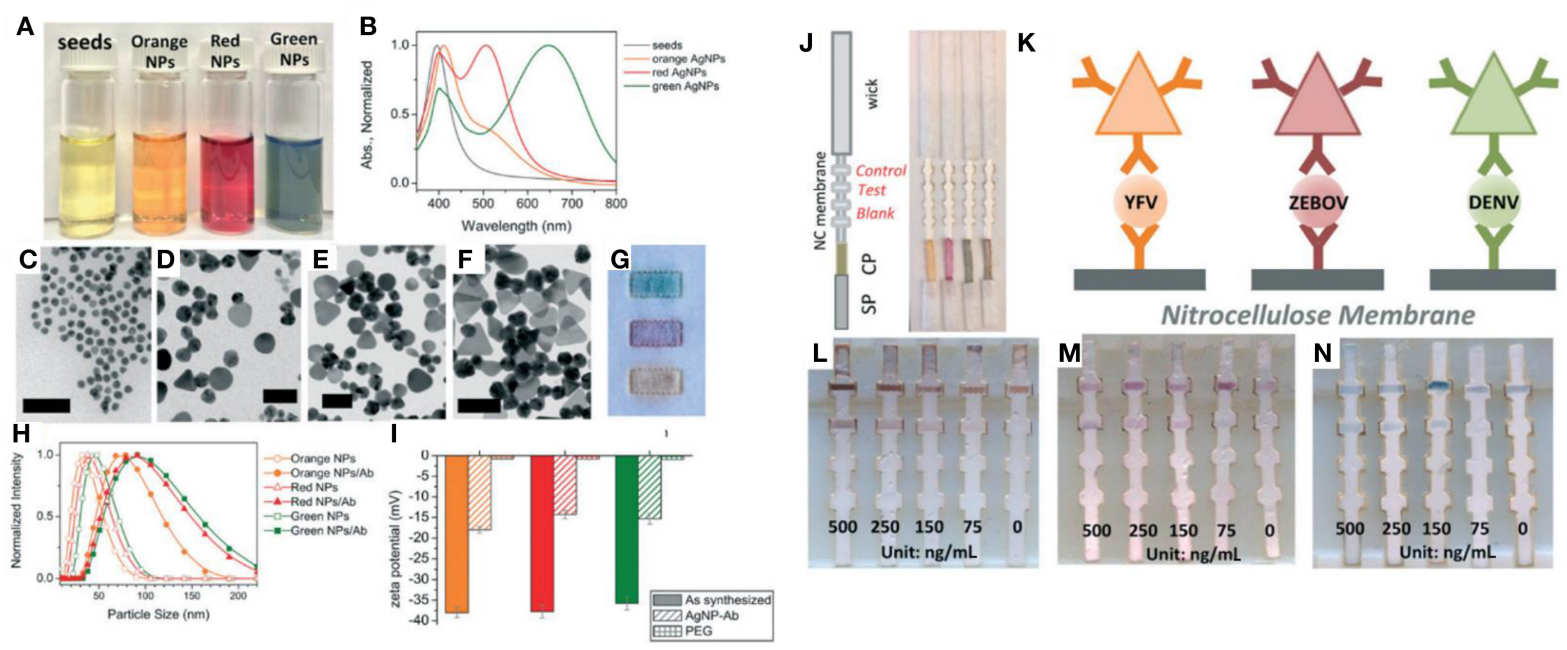
**An example of the use of AgNPs for multiplexed detection. (A)** Vials of AgNPs during stepwise growth and **(B)** their corresponding absorption spectra. TEM images of **(C)** Ag seeds, **(D)** orange AgNPs, **(E)** red AgNPs, and **(F)** green AgNPs. Scale bars: 50 nm. **(G)** Green, red, and orange (top to bottom) AgNPs on nitrocellulose paper. **(H)** DLS and **(I)** zeta-potential of AgNPs before and after antibody conjugation. **(J–N)** Illustration of the flow strips conjugate with the different antibodies and limit of detections of each biomarker. Yellow Fever (YFV), Zaire Ebola virus (ZEBOV), Dengue virus (DENV). Adapted from Yen et al. (2015) with permission of the Royal Society of Chemistry.

As a specific example of the use of AgNPs in the biomedical field, Yen et al. (2015) used AgNPs of different shapes and sizes to develop a rapid point-of-care diagnostic device for field-forward screening of severe acute systemic febrile illnesses such as Dengue, Yellow Fever, and the Ebola virus, respectively (see Figure 5). Another main use of AgNPs is their incorporation into products in the textile field. Wu et al. (2016), reported a simple and suitable fabrication of cotton fabrics with tunable colors, antibacterial capabilities, and self-healing superhydrophobic properties that can be used as protective clothing for working in moist and less-than-sanitary environments. This application consists of the deposition of branched poly(ethylenimine) (PEI) AgNPs and fluorinated decyl polyhedral oligomeric silsesquioxanes on cotton fabrics. Bollella et al. (2017) developed a green synthesis method to produce AgNPs by using quercetin (polyphenolic flavonoid). The AgNPs obtained were used to generate a novel third generation biosensor capable of measuring lactose in a large linear range, with high sensitivity and long-term stability.

Despite the promising economic benefit of the use of AgNPs and NPs in general, there are societal concerns associated with their use. For example, Miller and Wickson (2015) and Patenaude et al. (2015) discussed some barriers to accurate risk assessment and management of NPs and nanomaterials in general. These barriers include the lack of specific regulations for different types of NPs, the discrepancy between definitions, the lack of validated analytical methods and test protocols, the scarcity of reliable information about commercial use, and the lack of reliable exposure and toxicity data. Similarly, Hofmann et al. (2015) discussed the need for analytical methodology to accurately characterize NP morphology as well as the need for relevant toxicity assays in order to aid the development of regulations concerning inorganic NPs in the biomedical field. All of these developments, capital investment, research and development, legislative directives, and debate over regulatory approaches demonstrate the emergent role of NPs in technology, commerce, and society and show the importance of thoroughly evaluating environment, health and safety aspects associated with their use.

## Silver nanoparticles (AgNPs): possible impacts on environment, health and safety (EHS)

### Potential release of Ag and AgNPs in the environment

**Figure 6:**
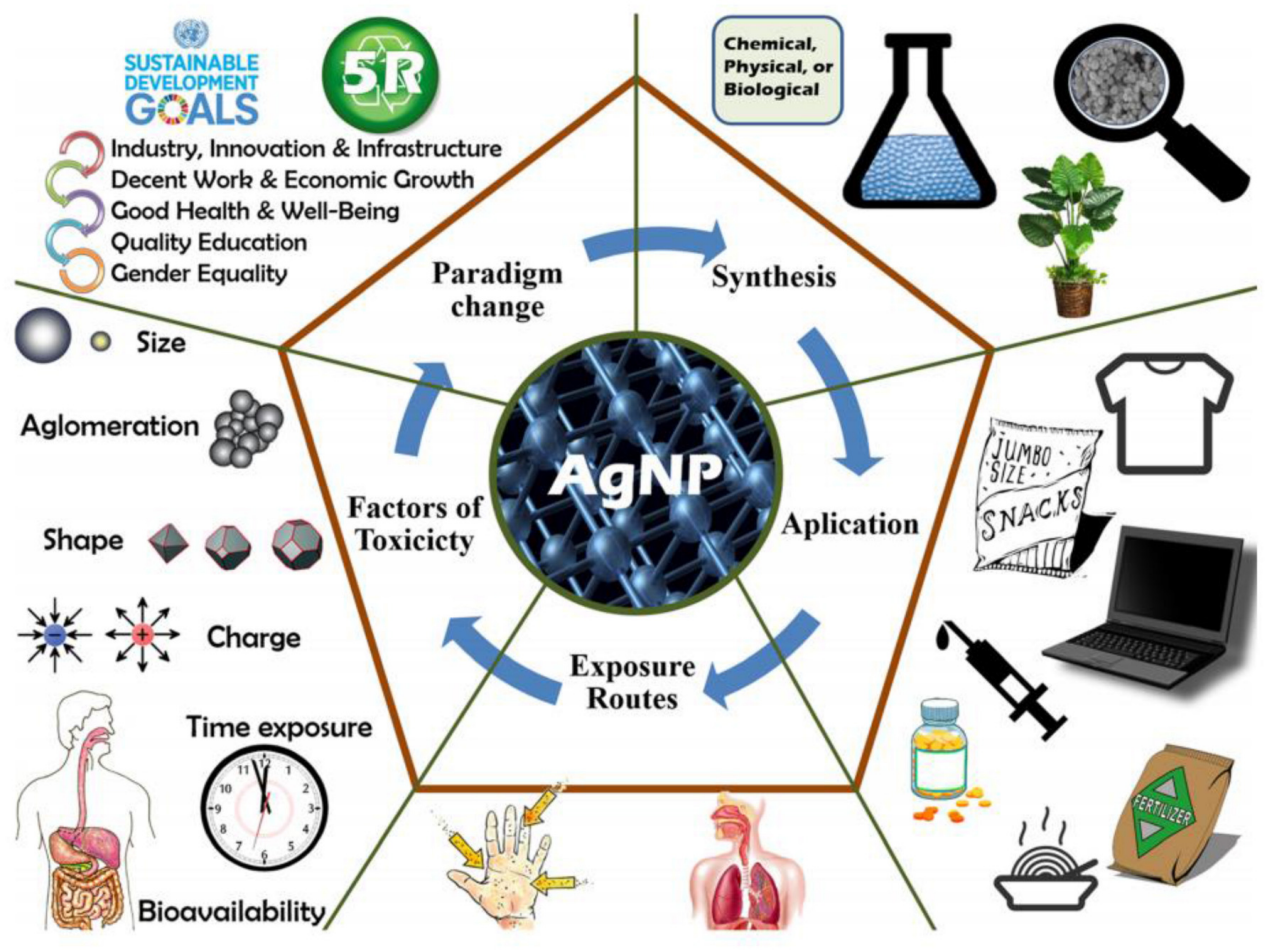
**Synthesis, application, routes of exposure, factors governing toxicology, and paradigm changes related to the AgNP production and use**. Reproduced from León-Silva et al. (2016) with permission of Springer.

With the increasing incorporation of nanomaterials into everyday consumer products, research efforts have been recently undertaken to understand the fate, transport, and subsequent effects of these NPs on the environment and higher organisms. Predictive models have been used in the U.S. and Europe to provide a prognostication of concentrations of AgNPs in surface waters, sewage treatment plant effluents, and sewage sludge; however, current data lack validation of the predictive modeling (Mueller and Nowack, 2008; Gottschalk et al., 2009). Further experimental modeling of assays is needed in order to implement standardized air and aquatic screening for AgNPs. The cache of studies related to the effects of AgNP production and use on the environment are still developing, however there is general agreement that AgNPs may be released into the environment during several routes and processes: Synthesis, during the manufacturing process and incorporation into products, recycling, and disposal (see Figure 6; Gottschalk and Nowack, 2011). One such study was conducted by researchers at the United States Consumer Product Safety Commission (Quadros et al., 2013), where the potential child AgNP exposure from a variety of consumer products (i.e., toys, fabric products, human milk storage bags, humidifiers, and accessories, etc.) was assessed by measuring the release of Ag^+^ and AgNPs into water, air, dermal wipes, orange juice, milk formula, and synthetic saliva, sweat, and urine. They were able to rank the products and categories on the basis of their potential for Ag bioavailability, from most likely to least likely to be a source of bioavailable Ag. Almost all the Ag released from fabric and toy samples was in the ionic form. They found that sweat and urine yielded the highest Ag^+^ release, while tap water had the lowest yield. While there are currently no guidelines for Ag in consumer products, their findings were significant as a proxy for release of Ag as AgNPs incorporated into various textiles, fabric, and cleaning products for antibacterial and purposes. Later, Mitrano et al. (2014) utilized a laboratory washing machine to simulate household laundering of textiles known to have undergone Ag and AgNP treatments to characterize and quantify total Ag release. Interestingly, conventional Ag treated fabrics yielded more total Ag and more nanoparticulate-sized Ag during fabric washing than the AgNP-treated fabrics. This was evidence that conventional forms of Ag precipitate to form nanosized Ag (complexes) and warrant careful considerations for regulatory action of nano-Ag as compared to conventional Ag forms. In fact, several other studies have focused on assessments and quantification of the release of Ag from AgNP-containing consumer products (Benn and Westerhoff, 2008; Kulthong et al., 2010; Von Goetz et al., 2013). Studies such as these allow researchers to understand the behavior of AgNPs in real-world scenarios as well as to aid risk assessments.

### Interaction of AgNPs and soil-plant systems

As residence times of NPs in soils and sediment generally exceed residence times in aquatic systems, the soil-environment has been shown to act as a major sink for AgNPs (Zhai et al., 2016). Increased interaction between terrestrial ecosystems and AgNPs are attributed to pathways that include on-site wastewater management systems, biosolids application, improper disposal, accidental spills, and the application of AgNPs-containing organic fertilizers and pesticides (Blaser et al., 2008; Anjum et al., 2013). Soil is representative of a complex matrix in which NPs can interact, and thus constitutes a great conduit toward understanding NP-physico-chemical behavior (Pan and Xing, 2012). Although limited studies exist concerning soil/AgNP interaction, modification of AgNP properties such as dispersibility, stability, agglomeration/aggregation, dissolution rate, aging, size, and surface area can occur through the interaction of soil environments and AgNPs, thus affecting their availability, retention, binding affinity, transport, and even toxicity to organisms (Bell and Kramer, 1999; Benn and Westerhoff, 2008; Geranio et al., 2009; Kim et al., 2010; Cornelis et al., 2012).

Ag and AgNP composites have found use in the control of various phytopathogens as well as for plant disease management (Liu et al., 2002; Park et al., 2006; Jo et al., 2009). Over the course of several studies, it was demonstrated that AgNPs were effective against plant fungus, providing evidence that AgNPs could serve a great purpose for controlling spore-producing fungal plant pathogens (Kim et al., 2009b; Jung et al., 2010; Lamsal et al., 2011a,b). While the latter studies demonstrate the benefit of AgNP soil treatment, AgNPs have also been found to have a deleterious effect, resulting in a drop in the metabolic abilities and diversity of necessary soil microbial populations (Jo et al., 2009). Hänsch and Emmerling (2010) identified that exposure to AgNPs of increasing concentration resulted in a significant decrease in microbial mass. A study by Zhai et al. (2016) demonstrated the potential for AgNPs of different shapes to disrupt the metabolic processes of natural soil microbial communities and also that soil microbes were more vulnerable to AgNPs on the smaller size spectrum.

### Interactions of AgNPs with biological media

The state of AgNPs is highly dependent upon their interaction with surrounding medium (Stebounova et al., 2011). Studies have provided compelling evidence that the interaction of AgNPs with biological media and biomolecules is complicated and can lead to particle agglomeration, aggregation, and dissolution (Stebounova et al., 2011; Argentiere et al., 2016). Investigations of the physical and chemical transformation of AgNPs allow more informative assessments of the potential of AgNPs to induce toxic responses (Park et al., 2013). Ionic strength, pH, and the presence of organic matter in biological and environmental media have been identified as some of the most critical factors that may contribute to the state and behavior of AgNPs. Stebounova et al. (2011) investigated the fate of AgNPs in two simulated biological fluids (artificial interstitial fluid and artificial lysosomal fluid) and concluded that the incubation of AgNPs in either simulated fluid led to both dissolution and precipitation of the NPs. AgNP-instability was attributed to the failure of the protective coatings on the NPs to prevent aggregation in the biological fluids (both of high ionic strength). In a similar way, citrate-stabilized AgNPs aggregated quickly in standard media recommended by the Organization for Economic Co-operation and Development (OECD) for *Daphnia magna* toxicity testing (Römer et al., 2011), where the high ionic strength of the media resulted in changes in organism exposure levels. Loza et al. (2014) studied the dissolution kinetics and nature of AgNPs after immersion in different media over 4,000 h. In their study, they hypothesized that the release of silver ions led directly to silver toxicity and confirmed this via cell culture-, microbiological-, and reactive oxygen species experiments. Researchers have also demonstrated that AgNPs in blood readily interact with surrounding biomolecules such as proteins and lipids, leading to the formation of protein coronas on the NP surface (Walczyk et al., 2010; Mahmoudi et al., 2013). On the other hand, it has been shown that the release of silver ions can be potentially suppressed by the addition of humic and fulvic acids, dissolved oxygen, natural and low salt sea water, and other organic matter (Liu and Hurt, 2010).

### *In vitro* and *in vivo* AgNP studies

In the past two decades, a large research effort has been devoted to the aspects of the toxicity of AgNPs, covering investigations of environmental fate, and including a plethora of *in vivo* and *in vitro* studies (Marambio-Jones and Hoek, 2010; Fabrega et al., 2011; Zhang et al., 2014). Comprehensive reviews have been compiled that detail the synthesis, application, subsequent routes of exposure, and toxicological mechanisms related to AgNP production and use (see Figure 6; León-Silva et al., 2016; Wen et al., 2016). Published cytotoxicity tests and *in vivo* assays lend limited evidence to claims that silver is carcinogenic in any tissue (U.S. Department of Health and Human Resources, 2010). However, a plethora of *in vitro* studies have provided evidence that AgNPs are not only transported into cells and internalized, but target endosomes and lysosomes (Asharani et al., 2009a; Luther et al., 2011), induce lung fibroblasts, impair the cellular membrane, cause DNA damage and genotoxicity, chromosome aberration, and apoptosis (Almofti et al., 2003; Asharani et al., 2009b; Yang et al., 2012; Jiang et al., 2013). Exposing A549 cells (human alveolar basal epithelial cells) to AgNPs resulted in not only reactive oxygen species generation, but reductions in cell viability and mitochondrial membrane potential (Chairuangkitti et al., 2013). Conversely, exposure to AgNPs at high concentrations (up to 6.25 μg/mL) caused not only apoptosis and oxidative stress but morphology changes in HT 1080 (human fibrosarcoma) and A431 cells (human skin/carcinoma) cells which became less polyhedral, more fusiform, shrunken, and rounded (Arora et al., 2008).

While there is evidence that AgNPs are toxic (Maurer and Meyer, 2016), the full mechanisms of toxicity are still not well-understood and research efforts should be devoted to gaining more clarity. The main drawbacks to establishing a systematic comparison of the current published studies are the lack of uniformity (in terms of size and shape) in the synthesis and the purification procedures of AgNPs, varying size distributions, coatings, and precursors, a lack of particle characterization, and the lack of implementation of validation with reference materials (Gliga et al., 2014; Gorham et al., 2014). Nonetheless, increased oxidative stress, apoptosis, and genotoxicity have been highlighted as the main *in vitro* outcomes of AgNP exposure (Kim and Ryu, 2013). These confounding differences in methodology have often lead to contradictory findings in *in vitro* studies. Studies that compare AgNPs of varying sizes show a greater toxic effect for particles of smaller diameter (Carlson et al., 2008; Braydich-Stolle et al., 2010). Oxidative stress has been the main link to the toxicity of AgNPs themselves (Kim et al., 2009a), but far more frequently, it is the dissolution of AgNPs that leads to toxic effects which makes an understanding of the ion release kinetics for AgNPs paramount (Foldbjerg et al., 2015). Burrell (2003) found that although inert in the presence of human tissues, metallic silver ionizes in the presence of bodily fluids and secretions, to release the biologically active Ag^+^ which has a high affinity to sulfydryl groups and other anionic ligands of proteins, cell membranes, and tissue debris (Burrell, 2003). Although Ag ion release has often been highlighted as the main cause of cytotoxicity and toxic effects, researchers find difficulty in determining the extent of the toxicity of AgNPs when Ag ions are also present in solution (the Ag ion induced effects often mask the effect of AgNPs at high metal ion concentrations). Foldbjerg et al. (2015) assert that research studies are still rife with confounding results the make ascertaining the cause of toxicity difficult to decipher. To date, the weight researchers must place on ion release when discussing AgNP toxicity is still a difficult concept to discern.

While AgNPs have been shown to be toxic to bacteria, hence their main use in the formulation of antibacterial products, significant evidence is present to support the toxicity of AgNPs to other organisms. Marambio-Jones and Hoek (2010) provide comprehensive evidence that AgNPs cause inactivity not only in bacterial cells, but also fungi, virii, and algae. AgNPs have also been found to be toxic to models such as zebrafish (Yeo and Yoon, 2009), *Drosophila melanogaster* (Ahamed et al., 2010), *Daphnia magna* (Scanlan et al., 2013), and *Caenorhabditis elegans* (Meyer et al., 2010 and Yang et al., 2014). Yeo and Yoon (2009) found that nano-silver ions penetrated the skin and blood tube of zebrafish larvae in the form of aggregates, while Ahamed et al. (2010) found that AgNPs induced heat shock, oxidative stress, DNA damage, and apoptosis in *Drosophila melanogaster*. Further, silver nanowires were not only toxic to *Daphnia magna*, but Scanlan et al. (2013) found that the surface coating of silver nanowires (AgNWs) was dramatically modified (as compared to pristine AgNWs) when extracted from the organism's hemolymph. In correlation with the effect that AgNPs have on soil and soil ecosystems, toxic effects have also been reported on a diverse range of soil invertebrates which include *Eisenia fetida, Enchytraeus albidus, Eisenia andrei, Porcellio scaber*, and *Folsomia candida* (Tkalec et al., 2011; Hayashi et al., 2012, 2013; Gomes et al., 2013; Schlich et al., 2013; Waalewijn-Kool et al., 2014; Gomes et al., 2015).

### Effects of Ag, AgNPs, and Ag constituents on human health

As can be seen in the aforementioned sections, AgNPs have been shown to have toxic effects to both *in vitro* and *in vivo models*, however there is a limited number of studies that report the impacts of AgNPs on human health (Korani et al., 2015); rather, the impact of silver is most often presented. Currently silver, present in the human body in low concentrations via inhalation of air particulate and through diet and drinking water, is considered relatively harmless to humans and is not regarded as toxic to the immune, cardiovascular, nervous, or reproductive systems (ATSDR, 1990; Lansdown, 2010). Even though the benefits of the Ag on human health are yet to be proven, colloidal silver suspensions are being incorporated into health supplements (Fabrega et al., 2011). Occupational health studies have found that long-term exposures to Ag have led to irreversible conditions such as argyria, wherein the skin turns bluish in color as a response to the accumulation of Ag in body tissues (Hill, 1941; Wadhera and Fung, 2005). It is worthy to note that the critical oral dosage that elicits this effect is not known and may vary from individual to individual. Silver and nanosilver accumulation in the skin, liver, kidneys, corneas, gingiva, mucous membranes, nails, and spleen are also possible (Rosenman et al., 1979; DiVincenzo et al., 1985; Hollinger, 1996; Sue et al., 2001; Wan et al., 1991). An extensive review of the exposure-related health effects of silver and silver related compounds was conducted by Drake and Hazelwood in 2005 and later by Lansdown in 2010 (Drake and Hazelwood, 2005; Lansdown, 2010). Studies have listed the liver as the primary organ for silver accumulation and elimination. Even though the majority of Ag-containing consumer products are designated for topical application, the risk of percutaneous absorption of silver is very low as the human epidermis is a relatively impenetrable barrier (the exception being dermal abrasions, wounds, and cuts). Lansdown (2006) also reasons that although there is an increasing use of Ag in silver thread and textile fibers, there has been no evidence of increased blood silver or accumulation of silver precipitates in the skin in chronic exposure and the risks of argyria in these cases have been deemed negligible. In the same vein, the toxic risks associated with silver ingestion are low, as most products releasing Ag ions for oral or gastrointestinal hygiene were removed from pharmacopeias and permitted lists in most countries, in light of the risks of argyria (Lansdown, 2010). More comprehensive studies and research efforts are necessary to clearly aid risk assessment, identify the toxic mechanisms of AgNPs and their toxicological effects where areas of human health are concerned.

## Synthesis and stabilization of silver nanoparticles (AgNPs) in liquid phase

Generally, the synthesis of NPs can be classified in two main categories: Top-down, where the procedure involves the use of bulk materials, such as metallic silver, that are reduced to form NPs using physical, chemical, or mechanical processes; or bottom-up, where the procedure requires starting from molecules, atoms, or ions to obtain NPs (Hornyak et al., 2008). Most NP synthesis approaches focus on bottom-up procedures, particularly in liquid phase media (Klabunde, 2001; Cunningham and Bürgi, 2013; Cushing et al., 2014; Majdalawieh et al., 2014) and nucleation theories and mechanisms have been extensively described by Cushing et al. (2014), Viswanatha and Sarma (2007), Finney and Finke (2008), Thanh et al. (2014), and Kettemann et al. (2016).

In recent years, the development of methods for the synthesis of AgNPs has been the subject of significant interest (Tran et al., 2013). Generally, AgNPs are synthetized in liquid phase using chemical methods such as: Classical reduction with citrate (Turkevich et al., 1951), reduction with NaBH_4_ (Lee and Meisel, 1982), reduction with gallic acid (Park et al., 2016), polyol synthesis (Kim et al., 2006), synthesis with organic solvents (Pastoriza-Santos and Liz-Marzán, 1999), as well as photochemical (Sun and Xia, 2002), electrochemical (Rodrıguez-Sanchez et al., 2000), and sonochemical methods (Jiang et al., 2004). However, despite the myriad of AgNP synthesis methods, few offer the capability to achieve shape and size control. The main impediments to the production of monodisperse, uniformly spherical AgNPs are the formation of secondary products (smaller and/or larger sizes) or undesirable shapes, such as nanorods, nanocubes, nanotriangles, nanodipyramids, and nanooctahedra (Shirtcliffe et al., 1999; Yang et al., 2011). Therefore, it is necessary to control and establish reaction conditions that facilitate reproducible synthesis of spherical NPs with uniform size distributions. In this context, some of the variables that can be tuned in the chemical synthesis process to control the size and shape of AgNPs are:

- the type and concentration of reducing agent (Dadosh, 2009) or stabilizing agent (Zhao et al., 2010);
- the addition of complexing agents (i.e., NH_3_) for removing precursor agents and decreasing particle size (Zhao et al., 2010);
- the addition of alkaline co-reducers using strong and/or weak reducing agents (Agnihotri et al., 2014).

Alternatively, other synthesis routes employ seed methods, where small NPs serve as seed or nucleation centers that allow control of the shape and particle size of the AgNPs (Jana et al., 2001; Pyatenko et al., 2007; Qu and Ma, 2012; Wan et al., 2013). The most common methods used for the synthesis of uniform and spherical AgNPs are summarized in Table 1.

**Table 1:** **Chemical methods for the synthesis of monodisperse and quasi-spherical AgNPs in liquid phase**.

**Precursor agent**	**Reduction agent**	**Capping agent**	**Some experimental conditions/results**	**References**
AgNO_3_	ascorbic acid	Glycerol/PVP	d ≈ (20 to 100) nm; temp ≈ 90°C	Steinigeweg and Schlücker, 2012
AgNO_3_	Na_3_Cit	Na_3_Cit/ TA	d ≈ (10 to 100) nm; temp ≈ 90°C	Bastús et al., 2014
AgNO_3_	EG	PVP/EG	d ≈ (10 to 80) nm; temp ≈ 160°C; t ≈ 4 h	Zhao et al., 2010
AgNO_3_	Na_3_Cit	Na_3_Cit	d ≈ (10 to 80) nm; temp ≈ b.p	Pyatenko et al., 2007
AgNO_3_	Na_3_Cit	Na_3_Cit	d ≈ (30 to 96) nm; temp ≈ b.p; pH ≈ 5.7 to 11.1	Dong et al., 2009
AgNO_3_	ascorbic acid	Daxad 19	d ≈ (15 to 26) nm; temp ≈ b.p	Sondi et al., 2003
AgNO_3_	NaBH_4_ or Na_3_Cit	Na_3_Cit	d ≈ (28 to 73) nm; temp ≈ b.p	Wan et al., 2013
AgNO_3_	Alanine/NaOH	DBSA	d ≈ 8.9 nm; temp ≈ 90°C, t ≈ 60 min	Yang et al., 2011
AgNO_3_	Na_3_Cit	Na_3_Cit/ TA	d ≈ (18 to 30) nm; temp ≈ 60°C to b.p; t ≈ 20 min	Dadosh, 2009
AgNO_3_	NaBH_4_/ Na_3_Cit	Na_3_Cit	d ≈ (5 to 100) nm; temp ≈ 90°C; pH: 10.5; t ≈ 20 min	Agnihotri et al., 2014
AgNO_3_	Oleic Acid	sodium oleate	d ≈ (5 to 100); temp ≈ (100 to 160)°C; t ≈ (15 to 120) min	Xu and Hu, 2012

*Na_3_Cit, Trisodium citrate; PVP, (C_6_H_9_NO)_n_ Polyvinylpyrrolidone; TA, (C_76_H_52_O_46_) Tannic acid; DBSA, (C_18_H_29_NaO_3_S) dodecylbenzenesulfonic acid; Daxad 19, Sulfonated Naphthalene Condensate (surfactant); b.p, boiling point*.

**Figure 7:**
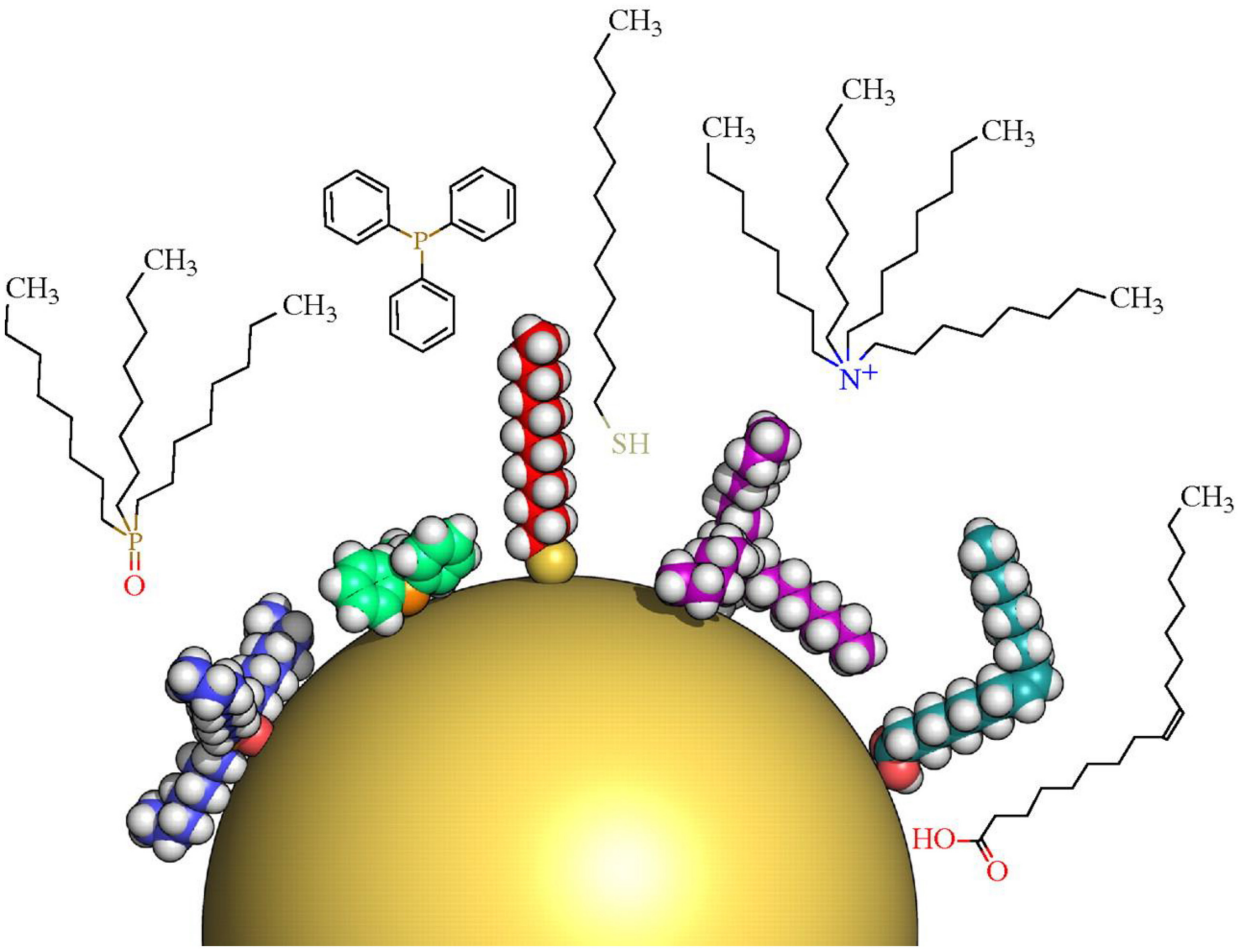
**An illustration of some selected surface chemistries and conjugation strategies that are applied to NPs**. Reproduced from Sperling and Parak (2010) with permission of the Royal Society.

Another important factor to consider for the synthesis of AgNPs in liquid phase is their subsequent stabilization. The stabilization of AgNPs is necessary for their compatibility across the range of applications described above (Kang and Haider, 2015) and will impact the interaction in the environment. In general terms, the stabilization processes decrease the NP surface energy making the colloidal system thermodynamically stable (Kraynov and Müller, 2011). Molecules and/or ligands bound to the NP surface not only control their growth during the synthesis process, but also aid in preventing aggregation; defined as a “particle comprising of strongly bounded or fused particles where the resulting external surface area may be significantly smaller than the sum of calculated surface areas of the individual components” (ISO/TS 80004-1, 2015), and agglomeration; defined as a “collection of weakly bound particles or aggregates or mixture of the two where the resulting external surface area is similar to the sum of the surface areas of the individuals components” (ISO/TS 80004-1, 2015; Manojkumar et al., 2016). The main mechanisms of interaction between these molecules and/or ligands with the surface of the NPs are mostly through chemisorption processes, electrostatic attractions, or hydrophobic interactions (Kraynov and Müller, 2011; Manojkumar et al., 2016). Figure 7 provides an illustration of functional groups with strong surface interactions with AgNPs (-SH, -NH, -COOH, -C = O) that allow for functionalization and further stabilization (Sperling and Parak, 2010).

**Figure 8:**
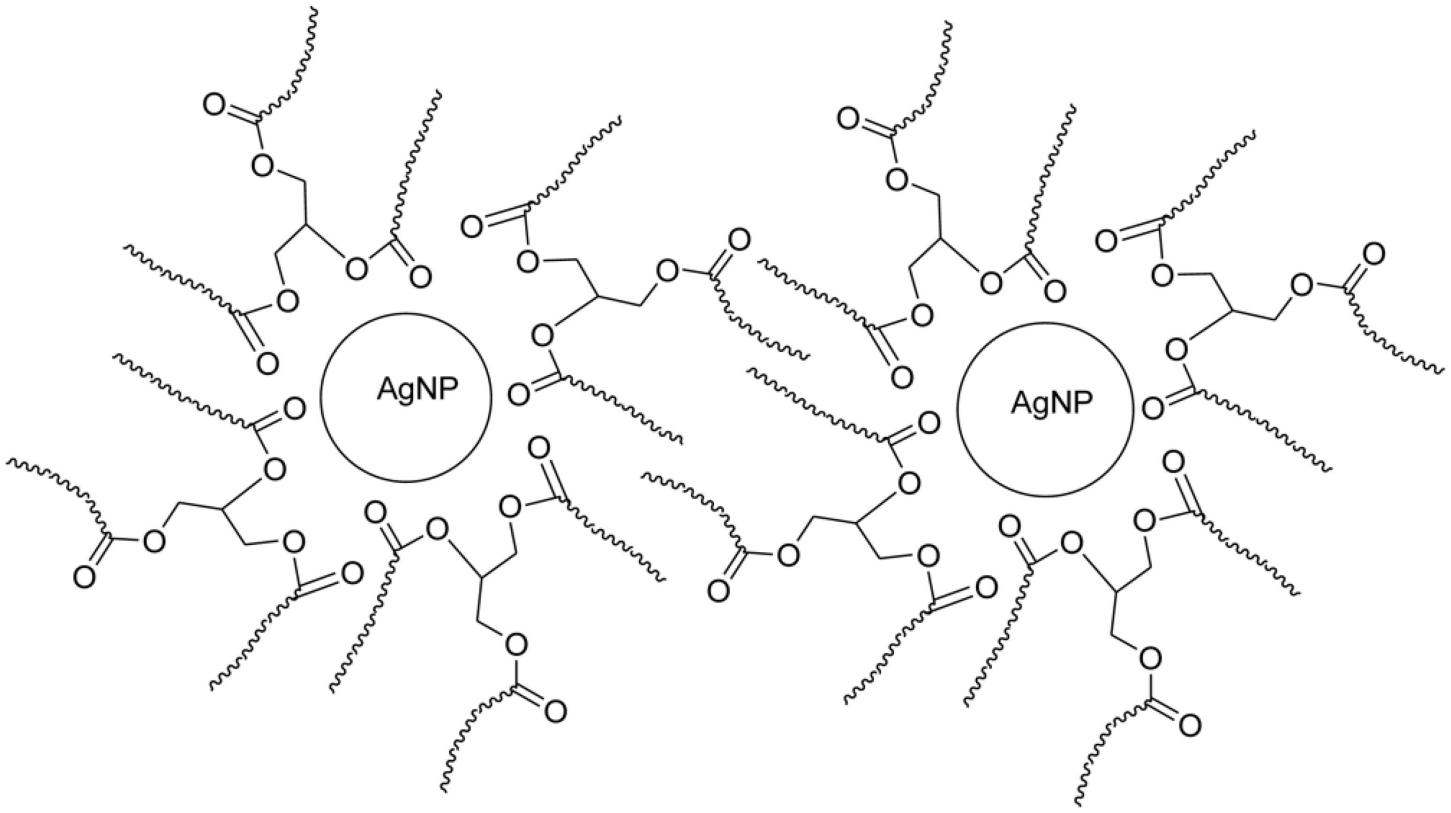
**Steric stabilization of AgNPs**. Reproduced from Zamiri et al. (2010) with permission of MDPI, Basel, Switzerland. CC BY 3.0.

Depending on the type of NP (i.e., the core material) and the dispersant solvent, the choice of a specific ligand can provide either higher or lower stability. Molecules with low molar mass have been used as stabilizing agents (Warner et al., 2000; Nath et al., 2010), however these types of molecules exhibit several limitations, including the easy desorption of ligands and the promotion of agglomeration and aggregation. (Van Hyning and Zukoski, 1998). Alternatively, synthetic polymers can be used for the stabilization of NPs. In this context, amphiphilic polymers have been employed to stabilize NPs (Mayer, 2001). Polymeric ligands tend to generate more contact points with the NP surface, creating better interaction ligand/surface interactions (adsorption) (Toshima and Yonezawa, 1998). On the other hand, hydrophilic polymer chain interactions generate external loops which can interact with the solvent and sterically stabilize NPs, (see Figure 8).

Stabilization will directly impact the physical and chemical properties of AgNPs, and subsequently may limit their applications. For example, studies have shown that AgNPs coated with polyvinylpyrrolidone (PVP) and polyethyleneglycol (PEG) have greater stability under environmental conditions than AgNPs stabilized using citrate (Lead et al., 2014). However, besides capping agents, storage temperatures are also critical to the stability of these materials. It has been shown that different storage temperatures can produce oxidation processes promoting unwanted shapes such as nanorods and nanoprisms or AgNP aggregation and/or agglomeration (Pinto et al., 2010). These processes are unintended in the synthesis of spherical and uniform size distributions of AgNPs; therefore, it is key to control the temperature of these colloidal systems to avoid (thermodynamically) the formation of such structures. Also, AgNPs can be modified and destabilized by photochemical reactions. Gorham et al. (2012) showed that AgNPs coated with citrate can be destabilized with UV radiation exposure. Other factors to take into account with regard to the destabilization of AgNPs are post-synthesis residues and incorrect purification procedures. For examples, high concentrations of remnant precursors and/or reducing agents in the liquid phase promote the transformation of AgNPs into new shapes such as nanorods, nanocubes, and nanotriangles (Murphy et al., 2001; Dadosh, 2009; Pinto et al., 2010). Additionally, pH plays an important role which is shown where amino acid-coated AgNPs have improved stability under acidic conditions (pH ≈ 3), eliminating the formation of agglomerates due to suppression of intermolecular interactions between solvent and ligand (Bayram et al., 2015).

Recently, it has been discovered that the use of biopolymers as a capping agents foster biocompatibility and safety from toxicological points of view (Jena et al., 2012). Specifically, different carbohydrates and their derivatives such as a guar gum (Vanamudan and Sudhakar, 2016), carboxymethyl cellulose (CMC) (Velusamy et al., 2016), dextran (Cakić et al., 2016), kappa-carrageen (Elsupikhe et al., 2015), sodium alginate (Chunfa et al., 2016), chitosan (Shanmugaraj and Ilanchelian, 2016), heparin (Kemp et al., 2009), and hyaluronic acid (Yahyaei et al., 2016) have been employed to stabilize AgNPs. Proteins have also been employed for the stabilization of AgNPs. Darroudi et al. (2011) provided a procedure for the sonochemical synthesis of AgNPs using gelatin as both a reducing and coating agent, obtaining very promising results in terms of sphericity and distribution of particles in the sub-10 nm range.

Furthermore, some studies demonstrate the good stability of capped biopolymer-AgNPs. Chen et al. (2008b) obtained highly stable AgNPs-CMC that showed no apparent change in their optical spectrum extinction when stored at 25°C for 58 days. Darroudi et al. (2011) determined that there is no change in optical extinction spectrum of AgNPs capped with chitosan/gelatin over a period of 4 months. Shanmugaraj and Ilanchelian (2016) later demonstrated that AgNPs capped with chitosan were stable for more than 4 months. All the studies described above, show that biopolymers can be used as capping agents and provide evidence for employing these macromolecules as stabilizing agents for NPs in liquid phase. Overall, the stabilization of AgNPs and other NPs in liquid phase is still considered a chemical challenge, mainly due to the complexity of some liquid media (biological, environmental, organic, etc.), environmental factors, and also due to the highly dynamic diffusion, sedimentation, agglomeration, and aggregation processes that AgNP experience which can reduce their entropy.

## Measurement and characterization of silver nanoparticles (AgNPs): A metrological approach

As previously mentioned, NPs constitute a focus of interest in nanoscience and nanotechnology (Kang and Haider, 2015; Sharma et al., 2015). Particularly, there is an interest in establishing controlled chemical (e.g., chemical composition of the core, surface chemistry, bulk element composition, internal/external chemistry of mixing state, and oxidation state) and physical properties (e.g., size, shape, number and mass concentration, surface area, total mass, crystallinity, morphology, and optical properties) of these nanoobjects. Moreover, due to advancements in the production and applications of nanomaterials, scientists are developing new, and adapting classic, analytical techniques for the detection, characterization, and quantification of NPs. An extensive discussion of the fundamentals and analytical capabilities of the most common techniques for the characterization of NPs (specifically metal, metal oxide and metalloid) has been thoroughly reviewed (Gunsolus and Haynes, 2015; Costa-Fernandez et al., 2016; Laborda et al., 2016; Majedy and Lee, 2016) and can be used for further consultation. The main current measurement techniques (MTs) for the characterization of NPs in general, and AgNPs in particular, and the requisite information they provide are listed in Table 2.

**Table 2:** **Common measurement techniques (MT) used for the characterization of NPs**.

**MT**	**Some properties**								**References**
	**Size/distrib**.	**Shape and morphology**	**Surface area**	**Surface chemistry**	**Chemical composition**	**Coating Chemistry**	**Chemical structure**	**Charge in suspension**	
AFM	•	•							Hoo et al., 2008
AES				•					Baer et al., 2010
ATR-FTIR						•			López-Lorente and Mizaikoff, 2016
BET			•						Brunauer et al., 1938; Schmid and Stoeger, 2016
CLS	•								Braun et al., 2011a
DMA	•								Mader et al., 2015
DLS	•							•	Tomaszewska et al., 2013
EDS					•				Patri et al., 2009
EELS							•		Hohenester et al., 2009
ETAAS					•				Hartmann et al., 2013
NMR						•			Liu et al., 2009; Marbella and Millstone, 2015
ICP-MS					•				Fabricius et al., 2014
PTA	•								Gallego-Urrea et al., 2011
SAXS	•								Li et al., 2016
SEM	•	•							Delvallée et al., 2015b
TOF-SIMS				•	•				Kim et al., 2015
sp-ICP-MS	•				•				Montaño et al., 2016
TEM	•	•							Pyrz and Buttrey, 2008
XPS				•					Baer et al., 2010

*AFM, Atomic Force Microscopy; AES, Auger Electron Spectroscopy; BET, Brunauer-Emmett-Teller method; ATR-FITR, Attenuated Total Reflectance Fourier Transfom-Infrared Spectroscopy; CLS, Centrifugal Liquid Sedimentation; DMAS, Differential Mobility Analysis; DLS, Dynamic Light Scattering; ET-AAS, Electrothermal Atomic Absorption; EELS, Electron Energy Loss Spectroscopy; EDS, Energy Disperse X-Ray Spectroscopy; ICP/MS, Inductively Couple Plasma Mass Spectrometry; NMR, Nuclear Magnetic Resonance; PTA, Particle Tracking Analysis; SAXS, Small-Angle X-Ray Scattering; SEM, Scanning Electron Microscopy; TEM, Transmission Electron Microscopy; SIMS, Secondary Ion Mass Spectrometry; TOF-SIMS, Time of Flight Secondary Ion Mass Spectrometry; spICP-MS, Single Particle Inductively Coupled Plasma Mass Spectrometry; XPS, X-Ray Photoelectron Spectroscopy*.

Advances in the characterization of NPs need to be accompanied with a standardized metrological approach (metrological traceability, estimation of the measurement uncertainty, use of standardized/validated methods, use of reference materials, participation in interlaborary comparisons) to assure the comparability of the measurements at the nanoscale. In others words, the measurements made using a metrological approach allow the establishment of extremely important variables in the quality of the measurements such as bias, precision and traceability to International System of Units (S.I). Consequently, it allows accurate and concrete conclusions of the chemical or physical property studied at the nanoscale. In the last decade, some institutions and standardization bodies have been working to establish standards, protocols, guidelines, and procedures for the correct measurement and characterization of NPs (see Table 3).

**Table 3:** **Representative standards, guides, and protocols developed in the recent years for the characterization of NPs**.

**MT**	**Type of MT**	**Organization or institution**		
		**NIST**	**ASTM**	**ISO**
AFM	Microscopy	Grobelny et al., 2009	ASTM E2859-11, 2011	–
ARS	Spectroscopic	–	–	ISO 20998-2, 2013
BET	Integral	–	ASTM E2864-13, 2013	ISO 9277, 2010
CLS	Centrifugation	–	–	ISO 13318-1, 2001
DMAS	Fractionation	Pease et al., 2010	–	ISO 15900, 2009
DLS	Spectroscopic	Hackley and Clogston, 2015	ASTM E2490-09, 2015; ASTM WK54872	ISO 22412, 2008
FT-IR	Spectroscopic	–	–	ISO/TS 1410
PTA	Microscopy	–	ASTM E2834-12, 2012	ISO 19430, 2016
SAXS	Spectroscopic	–	–	ISO 17867, 2015
SEM	Microscopy	Vladár and Ming, 2010	ASTM WK39049	ISO 13322-1, 2014
spICP-MS	Spectroscopic	Murphy et al., 2015	ASTM WK54613	ISO/TS 19590
TEM	Microscopy	Bonevich et al., 2010	–	ISO 29301, 2010; ISO 13322-1, 2014

*AFM, Atomic Force Microscopy; ARS, Acoustic Resonance Spectroscopy; BET, Brunauer-Emmett-Teller method; CLS, Centrifugal Liquid Sedimentation; DMAS, Differential Mobility Analysis system; DLS, Dynamic Light Scattering; FITR, Fourier Transfom-Infrared Spectroscopy; PTA, Particle Tracking Analysis; SAXS, Small-Angle X-Ray Scattering; SEM, Scanning Electron Microscopy; spICP-MS, Single Particle Inductively Coupled Plasma Mass Spectrometry; TEM, Transmission Electron Microscopy*.

While an extensive discussion of the measurement techniques and their analytical capabilities are beyond the scope of this review, we will focus on the metrological aspects of some of the most important measurement techniques for the characterization of AgNPs. In this context, microscopy techniques are extremely powerful analytical tools for the characterization of AgNPs. For example, MacCuspie (2011) used Atomic Force Microscopy (AFM) with the goal of exploring the stability of AgNPs capped with citrate and bovin serum albumin (BSA) in solvents with different electrolyte concentrations and pH conditions. Their AFM results, accompanied with measurements of ultraviolet-visible spectroscopy (UV-Vis) and dynamic light scattering (DLS), showed how the stability of the AgNPs are highly affected by different factors (pH, electrolytes concentrations, and capping agent). Also, they demonstrated how different MTs such as AFM, UV-Vis, and DLS, can be used for evaluating the stability and characterizing these colloidal systems. Transmission electron microscopy (TEM) and scanning electron microscopy (SEM) are other microscopy techniques widely used in the characterization of AgNPs in the metrological field. Klein et al. (2011b) used SEM and TEM in order to characterize and establish the particle size and size distribution of its representative test material, NM-300 (see definition of representative test material, RTM, in the next section of this document). The use of complementary MTs such a UV-Vis, graphite furnace atomic absorption (GFAAS), and inductively coupled plasma optical emission spectrometry (ICP-OES) were used to study stability, the release of ionic silver, and to quantify the total silver mass of this RTM, respectively. Recently, Verleysen et al. (2015) used TEM for the measurement and the validation of 23 dimensional and morphological parameters (diameter, perimeter, central distance, shape factor among others) of AgNPs, providing the measurement uncertainty of these parameters. In the same context, Dudkiewicz et al. (2015) reported the use of electron microscopies (SEM and TEM) for the characterization of AgNPs spiked into two different food matrices (chicken paste and tomato soup). Their study has generated a key metrological input in the determination of particle size in a complex matrix (food) by electron microscopy techniques, because they assessed the impact of different sources of uncertainty such as sampling, sample preparation prior to imaging, and image analysis in the total uncertainty of the particle size determination.

In general terms, microscopic techniques have been a focus of attention for the metrological characterization of NPs. For example, in recent years the effect of different substrates on the determination of the particle size has been studied by AFM (Delvallée et al., 2015a,b). Also, different detector systems such as darkfield, brightfield (Buhr et al., 2009; Klein et al., 2011b) and energy dispersive X-ray detectors (EDS) (Hodoroaba et al., 2016) have been used in SEM measurements for the determination of particle size, size distribution, and chemical surface of different NPs. Additionally, systematic procedures for the generation of an unbiased random image collection, validation of size, shape, and surface topology measurements and for the evaluation of measurement uncertainty using TEM have been proposed by De Temmerman et al. (2014). Moreover, various statistical criteria have been established to select the correct number of particles (population) for the determination of the size and size distribution of NPs using TEM (Song et al., 2009; Rice et al., 2013). Other techniques such as dynamic light scattering (DLS), (Takahashi et al., 2008; Kwon et al., 2011), centrifugal liquid sedimentation (CLS), (Braun et al., 2011a), and nanoparticle tracking analysis (NTA), (Hole et al., 2013), are currently being implemented in the metrological field for the characterization of different varieties of NPs (metal, metal oxide, and metalloid NPs). A good example is the development of RMs in the nanoscale, where the combination of multiple methods is necessary to assign and characterize the different properties of these materials.

In the specific case of AgNPs, many of the MTs described above can be used for the characterization of their chemical and physical properties. For example, MacCuspie et al. (2011), report the use of multiple MTs such AFM, TEM, DLS, NTA, and ultrasmall angle X-ray scattering (USAXS) for the physico-chemical characterization of AgNPs. The same group also discuss the different results obtained by these MTs in the determination of the size, size distribution and agglomeration of the AgNPs. Martin et al. (2014) used USAXS and TEM to understand and study the dissolution, agglomeration, morphology, and stability of AgNPs exposed under different acid concentration (HNO_3_). Moreover, they used UV-Vis and DLS to investigate the stability of AgNPs in strong acid media and evaluated the morphology the AgNPs coated with BSA. Murphy et al. (2015) established a protocol for the determination of mean nanoparticle size (equivalent spherical particle diameter), number based size distribution, particle number concentration, and mass concentration of ions in an aqueous suspension of AgNPs using single particle inductively coupled plasma mass spectrometry (spICP-MS). These are just some examples of the different MTs that can be used for the characterization of AgNPs. Finally, all these techniques can be employed in concert toward one of the most important task in the chemical metrology field: The development of reference materials (see Table 4). A good example of this is the multimethod approach used by NIST in the development of the NIST RM 8017 PVP-coated AgNPs (NIST, 2015d). In their investigation report, AFM, TEM, USAXS, and DLS was used by NIST researchers to determinate the particle size of this nano-object. It is important to mention that the determination of the particle size of NPs is method dependent, and as a result of this, NIST attempted to characterize its RM using different MTs. Other MTs such as isotopic dilution mass spectroscopy (IDMS), asymmetric-flow field-flow fractionation (AF4), ICP-MS, UV-Vis, and spICP-MS have been used to characterize important properties in the RM including the silver mass content, elemental impurities, absorbance spectrum and others.

**Table 4:** **NPs reference materials and certified reference materials developed in the recent years**.

**Material**	**Property measured**	**Form/quantity**	**Value and uncertainty**	**MTs used**	**NMI(id)**	**Proposed uses**	**References**
AuNPs^a^ (RM)	Particle size	LS/5 ml	(8.5 ± 0.3) nm	AFM	NIST^f^(RM 8011)	Instrument calibrations, evaluation of *in vitro* assays (bioassays), interlaboratory comparison	NIST, 2015a
			(9.9 ± 0.1) nm	SEM			
			(8.9 ± 0.1) nm	TEM			
			(11.3 ± 0.1) nm	DMA			
			(8.5 ± 1.8) nm	SAXS			
AuNPs^a^(RM)	Particle size	LS/5 ml	(24.9± 1.1) nm	AFM	NIST^f^(RM 8012)	Instrument calibrations, evaluation of *in vitro* assays (bioassays), interlaboratory comparison	NIST, 2015b
			(26.9 ± 0.1) nm	SEM			
			(27.6 ± 2.1) nm	TEM			
			(28.4 ± 1.1) nm	DMA			
			(28.6 ± 0.9) nm	DLS (173°)			
			(26.5 ± 3.6) nm	DLS (90°)			
			(24.9 ± 1.2) nm	SAXS			
AuNPs^a^(RM)	Particle size	LS/5 ml	(55.4 ± 0.3) nm	AFM	NIST^f^(RM 8013)	Instrument calibrations, evaluation of *in vitro* assays (bioassays), interlaboratory comparison	NIST, 2015c
			(54.9 ± 0.4) nm	SEM			
			(56.0 ± 1.5) nm	TEM			
			(56.3 ± 1.4) nm	DMA			
			(56.6 ± 0.9) nm	DLS (173°)			
			(55.3 ± 3.6) nm	DLS (90°)			
			(53.2 ± 1.2) nm	SAXS			
						
AgNPs^b^(RM)	Particle size	DS/ ≈ 2 g	(70.1 ± 6.0) nm	AFM	NIST^f^(RM 8017)	Benchmark and evaluation of potential EHS	NIST, 2015d
			(74.6 ± 3.8) nm	TEM			
			(67.9 ± 0.5) nm	USAXS			
			(105.6± 4.6) nm	DLS			
	Mass value		(2.162 ± 0.020)^f^ mg	IDMS			
AgNPs^c^(CRM)	Particle size	LS/5 ml	d_10_(12.0 ± 1.9)^d^ nm d_50_(18.5 ± 2.5)^d^ nm	SAXS	BAM^g^(BAM N001)	Used as standard material for measurements and toxicological test	Menzel et al., 2013
			d_90_(18.5 ± 2.5)^d^ nm d_10_(6.9 ± 1.9)^e^ nm				
			d_50_(12.6 ± 2.5)^e^ nm				
			d_90_(19.4 ± 2.5)^e^ nm				
SiO_2_-NPs(CRM)	Particle size	LS/10 mL	(19.0 ± 0.6) nm	DLS	IRMM^h^(ERM FD100)	Evaluated, Instrument and method performance	Braun et al., 2011b
			(20.1± 1.3) nm	CLS			
			(19.4 ± 1.3) nm	TEM			
			(21.8 ± 0.7) nm	SAXS			
SiO_2_-NPs (CRM)	Particle size	LS/9 mL	(42.1 ± 0.6) nm	DLS	IRMM^h^(ERM FD 304)	Evaluated, Instrument and method performance	Franks et al., 2012
			(33.0 ± 3.0) nm	CLS			
PS (CRM)	Particle size	LS/5 mL	(60.39 ± 0.63) nm	DMA	NIST^f^SRM 1964	Calibration/validation of particle sizing instruments	NIST, 2014a
PS (CRM)	Particle size	LS/5 mL	(60.39 ± 0.63) nm	DMA	NIST^f^(SRM 1963a)	Calibration/validation of particle sizing instruments	NIST, 2014b
TiO_2_(CRM)	Specific Surface Area	PPS	(55.55 ± 0.70) m^2^g^−1^	MP-BET	NIST^f^(SRM 1898)	Benchmark and evaluation of potential EHS	NIST, 2012
			(53.85 ± 0.78) m^2^g^−1^	SP-BET			

*CRM, Certified Reference Material; DS, Dry Solid, EHS; Environmental, Health, and Safety Risks; RM, Reference Material; LS, Liquid Suspension; PPS, Powder and Porous Solid*.

a *citrate-stabilized AuNPs in an aqueous suspension*.

b *lyophilized polyvinylpyrrolidone (PVP)-coated AgNP*,

c *AgNPs stabilized against aggregation using polyoxyehylene glycerol trioleate, polyoxiethylene sorbitan monolaurate*,

d *The d_10_, d_50_, and d_90_ values are specific particle diameters (volume weighted) that correspond to 10,50, and 90% of the total particles in cumulate undersize distribution*,

e *The d_10_, d_50_, and d_90_ values are specific particle diameters (number-weighted) that correspond to 10,50, and 90% of the total particles in cumulate undersize distribution*,

f *Expanded uncertainties, U, calculated as U = ku_c_, where u_c_ is intended to represent, at the level of one standard deviation, the combined standard uncertainty calculated according to the ISO/JCGM Guide (BIPM et al., 2008). The coverage factor, k, for 95 % expanded uncertainty intervals is based on a t multiplier with the appropriate associated degrees of freedom*,

g *Expanded combined uncertainty consisting of contributions from method repeatability, measurement setup geometry, method bias, possible but undetected inhomogeneity and instability, and the model used, in particular binning, expanded by a factor or k = 2 corresponding to a confidence level of ~95%*,

h *The certified uncertainty is the expanded uncertainty with a coverage factor k = 2 corresponding to a level of confidence of about 95 % estimated in accordance with ISO/IEC Guide 98-3:2008 (ISO/IEC Guide 98-3, 2008)*.

Despite this, further advancements are necessary to work toward improving the measurement and characterization of AgNPs and NPs in general, as many analytical techniques are still hampered with limitations (especially at the small end of the nanoscale range, i.e., sub-10 nm). In the specific case of AgNPs, the simultaneous determination of ionic silver and AgNPs in colloid suspensions still present an analytical challenge for most of the MTs. This aspect is solved partially by techniques like spICP-MS, however limitations such as limit of detection (LOD) and the overlap of ionic silver and AgNPs signals still obstruct the characterization by this technique in some cases. On the other hand, a large number of nanotoxicological and environmental studies lack a metrological approach, leaving out important metrological tools that enable the comparability and reproducibility of results. Such tools include standardized/validated methods, use of reference materials, and the estimation of the measurement uncertainty in the nanoscale. The studies described above reflect the continued importance of the development of robust, comparable, analytical methodology in order to achieve improvement of measurement in the nanoscale.

## Development of nanoparticle reference materials (RMs) in the nanoscale

**Figure 9:**
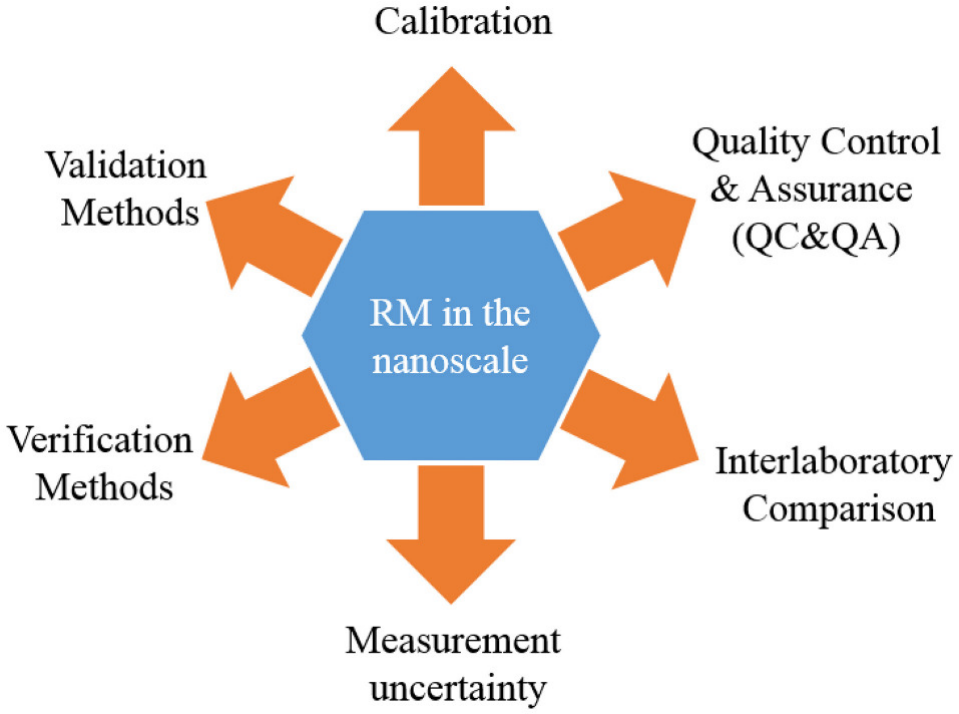
**Uses of reference materials in the nanoscale**.

Advances in nanoscience create demand for improvement in measurement capabilities. Therefore, quantitative measurements, stable instruments (in terms of drift, instrumental noise, sensitivity, and LOD), measurement protocols, and reference materials (RMs) are metrological mechanisms necessary for the advancement and consolidation of reliable and traceable measurements in this field (Picotto et al., 2009). Specifically, RMs play an integral role in the improvement and quality assurance of measurements in the nanoscale (see Figure 9). For example, (Montoro Bustos et al., 2015), reported the first post hoc interlaboratory study using the NIST RM 8012 (AuNPs, nominal 30 nm diameter) and RM 8013 (AuNPs, nominal 60 nm diameter) to evaluate the independent particle size measurements made by researchers in academia, government, and industry using single particle inductively coupled plasma mass spectrometry (spICP-MS). Meli et al. (2012), used different RMs, specifically the NIST RM 8011 (AuNPs, nominal 10 nm diameter), NIST RM 8012 (AuNPs, nominal 30 nm diameter), NIST RM 8013 (AuNPs, nominal 60 nm diameter), and IRMM-304 (Colloidal Silica Reference Material developed by the Institute for Reference Materials and Measurements, IRMM) in order to validate the measurement results and uncertainty estimations reported by various European Metrology Institutes using different MTs (AFM, DLS, SAXS, SEM). Others examples are consistent in demonstrating the critical role of RMs in improving the comparability of the measurements in the nanoscale (Roebben et al., 2011; Braun et al., 2012). However, in this context it is important to define what is considered a RM. According to ISO, a RM is a “material, sufficiently homogeneous and stable with respect to one or more specified properties, which has been established to be fit for its intended use in a measurement process” (ISO/Guide 30, 2015). In a practical way, a RM is a material with enough trueness to be used as a standard in a measurement. Subsequently, a certified reference material (CRM) is defined by ISO as a “reference material characterized by metrological valid procedure for one or more specified properties, accompanied by a certificate that provides the value of the specified property, its associated uncertainty and a statement of metrological traceability” (ISO Guide 30, 2015). The term “CRM” introduces two main metrological concepts: Measurement uncertainty and metrological traceability. Therefore, the basic difference between a RM and CRM is the status of the property values assigned to the material (Roebben et al., 2013). In the nanoscale, these definitions have the same meaning, nevertheless, the complexity of the systems and measurement capabilities at the nanoscale makes the development of CRMs more challenging because many of the measurands are method-defined making it difficult to establish a clear link to the SI. The measurement of chemical and physical properties of sub-10 nm nano-objects is a challenge for most analytical techniques and, reactivity, aggregation, agglomeration, and interactions between the dispersant medium add more complexity to the measurement system resulting increase in the uncertainty of the measurement.

For all these reasons, in recent years there has been a strong interest in developing NP RMs in the nanoscale, since they can shed new light not only on the impact of nanomaterials with respect to EHS, but also on ways in which the quality of measurements in the nanoscale can be improved or quality-assured (Hansen et al., 2007; Stefaniak et al., 2013). Aitken et al. (2008), established a priority list of candidate materials for the production of nanotoxicology RMs. This list consisted of several nanomaterials such as a carbon black, single and multiwalled carbon nanotubes (SWCNT/MWCNT), fluorescent polystyrene, combustion-derived NPs, TiO_2_-NPs, ZnO-NPs, AgNPs. Others materials such as AuNPs, CeO_2_-NPs, SiO_2_-NPs, ceramics, and nanoclays were identified also as a potential RMs. Stone et al. (2010), evaluated which of these materials were suitable for employment in ecotoxicological studies. They identified TiO_2_-NPs, polysterene beads labeled with fluorescent dyes, and AgNPs, as materials that would be useful to produce test- or reference materials. A comprehensive approach for the prioritization of materials that can be developed into reference materials was made recently by Stefaniak et al. (2013), where a list of 25 individual nano-objects was generated with scientific interest for the generation of RMs for risk assessment. In particular, they highlighted NPs such as CeO-NPs, SiO_2_-NPs, TiO_2_-NPs, ZnO_2_-NPs, AuNPs, and AgNPs. Table 4 lists RMs developed in the recent years by different National Metrology Institutes (NMIs). As can be seen, the proposed purposes of these RMs ranges from instrumental calibration to the evaluation of potential EHS risks.

Despite the identification and prioritization of materials to be developed as RMs in the nanoscale, the properties required to be assigned or certified have a huge importance in the development of a new RM. Composition (elemental/molecular), surface area, particle size, particle size distribution, morphology/shape/form, surface chemistry, agglomeration/aggregation state, crystal structure, and surface charge are frequently suggested to be value-assigned in RM candidates. Additionally, some challenges still remain from the metrological standpoint in regards to certifying properties in the nanoscale. Many of these properties are only broadly defined or qualitative (i.e., aggregation and agglomeration), limiting the possibility of assigning a reference value (Stefaniak et al., 2013). As mentioned above, many of these properties are method-dependent; for instance, the determination of the particle size in a reference material in the nanoscale is usually made using different MTs such as DLS, AFM, TEM, SEM, NTA, and others which rely on different measurands (Kestens et al., 2016). The production of RMs requires the establishment of validated methods with a full estimation of the uncertainty sources that have been involved in the measurement (ISO/Guide 34, 2009). This aspect may seriously hinder the development of RMs, because as previously mentioned, only a few standardized procedures for the characterization of properties of NPs are available, the majority of which are focused on the determination of dimensional properties such as particle size and particle size distribution (see Table 3).

Matrix plays an important role in the production and certification of NP RMs. Grombe et al. (2015), described the feasibility of the development of RMs for the detection of AgNPs in food matrices. Their results indicate significant differences in particle size when the AgNPs are dispersed in meat materials in comparison to water suspensions. They also reported difficulties in the development of efficient methods for the detection of AgNPs, principally due to AgNP reactivity being higher in comparison to more stable NPs (e.g., metal oxides like TiO_2_). Furthermore, another important factor to consider in the production of RMs at the nanoscale, is the form of the nanomaterial. Linsinger et al. (2011), discussed in detail two different forms (states of matter) that are conceivable for NP RMs: Suspensions of particles and dry powders. In suspension, NPs have better motion (promoted by Brownian Motion and diffusion process), producing an easier dispersion and homogenization of the material. However, this can promote the interaction with other molecules or even promote interaction between the NPs (aggregation, agglomeration, Ostwaltd ripening, or coarsening). For example, Gorham et al. (2014) demonstrate AgNPs suspensions capped with citrate lose their physical and chemical integrity by oxidation process and oxidation process followed by photoreduction. On the contrary, in powder form, NPs are more stable, essentially because the chemical changes only progress by diffusion, which is a rather slow process in this state of mater. To promote long-term stability, some dry powder RMs are stored in inert atmospheres, preventing the chemical degradation of the materials (Hornyak et al., 2008). MacCuspie et al. (2013), stabilized AgNPs in excess PVP and then lyophilized the formulated AgNPs to produce a cake of NPs that can be reconstituted simply by adding water. This approach resulted in a practical way to eliminate chemical changes of the AgNPs, conserving the particle size within the shelf-life required for a RM and was used in the development of the NIST RM 8017 Polyvinylpyrrolidone Coated Silver Nanoparticles (Nominal Diameter 75 nm) (NIST, 2015d). A drawback of the use of dry powder RMs is the possible need for redispersion protocols (Linsinger et al., 2011) to ensure that a homogeneous suspension is formed. This can be problematic, especially in the case of users with limited experience or expertise in sample preparation procedures and could generate a bias which is not intrinsic to the property certified. On the other hand, NP RMs in suspension (liquid phase) are characterized with respect to homogeneity and are easier to use. However, as was previously discussed that NPs in liquid phase (colloidal suspensions) need to be correctly functionalized in order to prevent their destabilization, which can create issues in ensuring long-term stability. In this aspect, Orts-Gil et al. (2013) pointed out that the development of functionalized, colloidal, stable RMs, may improve comparability between results across different laboratories, and provide convenience and feasibility in establishing multi-parametric RMs for engineered NPs.

It is important to mention that the development of a NP RM is an arduous process that involves many technical and production requirements (for example, production planning, production control, material storage, material processing, data acquisition, data evaluation, and in the case of CRMs, establishing metrological traceability, etc.) (ISO Guide 34, 2009). Recently, a new term has been proposed: “Representative Test Material (RTM).” RTMs will serve to cover gaps in the availability of NP RMs (Roebben et al., 2013). Specifically, a RTM is defined as “a material from a single batch, which is sufficiently homogeneous and stable with respect to one or more specified properties, and which implicitly is assumed to be fit for its intended use in the development of test methods which target properties other than the properties for which homogeneity and stability have been demonstrated (ISO/TS 16195, 2013).” In the recent years, the Organization for Economic Co-operation and Development (OECD), in conjunction with the European Joint Research Center (JRC), has worked on the development of a wide range of RTMs to support nanomaterial research and development. Some examples of the RTMs developed at this moment are illustrated by Singh et al. (2011), Klein et al. (2011a), Rasmussen et al. (2014), Roebben et al. (2015) and on the website of the JRC (JRC, 2016).

## Outlook and perspectives

Concepts, definitions, and terminology in nanoscience and nanotechnology are currently changing in response to increased research efforts and the extraordinary growth that this area has experienced in the last two decades. Several factors (economic, social, and environmental) are promoting the establishment of robust and well-founded terminology that contribute to building sensible legislation and regulation. However, the development and consistent implementation of defined “nano” terms represent a tremendous challenge.

Regardless of the difficulty in implementing regulation and legislation, a large number of scientific and technological applications and commercial products already incorporate NPs into their design. Particularly, AgNPs have been listed as a one of the most used nano-objects in commerce, mainly due to its versatile properties (catalytic, optical, engineering, electrical, biomedical, among others). The promising economic and technological landscape of NP applications emphasizes the concern regarding possible environmental, health, and safety (EHS) risks of these materials. In the last 10 years, toxicological, ecotoxicological, and genotoxicogical effects of AgNPs have been indicated in many studies. So far, the state of the art of nano-EHS research is promising and evolving, but its development is still limited in comparison to the exponential growth of new applications and products that incorporate NPs into their formulations. Moreover, the understanding of toxicity mechanisms, long-term accumulation effects, and dose-response relationship is still in its infancy. As a result, more studies will center around making accurate assessments of the implications and impacts of the production of AgNPs on EHS over the next years. To address these challenging tasks, more studies and results derived from rigorous *in vitro* and *in vivo* studies (e.g., bioaccumulation and bioavailability) will be necessary in order to elucidate the true impact of these materials. These studies will provide a scientific and technical basis for building worldwide consensus on regulation. Also, a concerted multidisciplinary effort must be continued to capitalize on initial findings in order to advance the investigation of relevant environmental scenarios.

Other efforts should be made in the area of NP synthesis and stabilization. Nowadays, among the great variety of chemical routes for the synthesis of AgNPs, only few enable control of the production of NPs with sufficient homogeneity of size and shape, both key parameters and highly demanded in the development of new applications. Besides that, the development of new synthesis routes that are much more efficient and use green synthesis approaches present emerging strategies to make the production of AgNPs more sustainable and environmentally friendly. Additionally, the use of biopolymers such as proteins, carbohydrates, and other types of macromolecules as stabilizing and functionalizing agents can improve the long term stability of the AgNPs in liquid phase and increase biocompatibility with environmental and biological systems. So far research into the stabilization of AgNPs using biopolymers is not sufficiently advanced to establish a clear stabilization mechanism using these coating agents. The behavior of this type of functionalized AgNPs under various conditions or factors that can compromise stability such as pH, temperature, UV radiation, etc., has yet to be studied. Given the above concerns, it is necessary to perform in-depth investigations of synthesis routes using biopolymers that control the shape, size, and stability of AgNPs.

With regard to the metrological field, the characterization of NPs is still considered a challenge because some measurement properties are method dependent, which hampers the comparison of values obtained from different measurement techniques. Continuous efforts have been made by the scientific community to standardize measurement protocols. In fact, some protocols, technical standards, and procedures have already been generated by different international organizations (e.g., ISO/TC 229 and ASTM E56) in order to provide more suitable and robust methods. Specifically, these efforts have focused mainly on the dimensional properties (size, shape, and distribution) of nanomaterials. However, it is necessary to develop/implement analytical techniques to extend the NP characterization capabilities toward the measurement of other important properties such as surface chemistry, chemical structure, and chemical composition. Moreover, the ability to provide traceability to the SI at the nanoscale level has also proven to be quite a challenge. Some of the MTs may be directly or indirectly linked to the S.I., however many of these MTs provide semiquantitative and/or qualitative measurements that are not metrologically traceable. This is a limiting factor in areas such as nanotoxicology, ecotoxicology, and biomedical applications where these properties like surface charge, hydrophobicity, and agglomeration state play critical roles. Finally, the development of NP RMs is crucial to providing sound metrological tools for industry and the scientific community implementation to evaluate their measurement capabilities. However, presently the availability of NP RMs is quite limited because of the technical complexity that is involved the production of these materials. Though more RMs have been developed in recent years, in many cases RMs are not available for relevant measurands and to cover the myriad of scenarios where nanomaterials are being currently applied. It is expected that in the next few years more RMs and RTMs will be released in order to provide comparability and to assure the quality of measurements in the nanoscale.

## Author contributions

BC reviewed the literature and wrote the manuscript text. MJ wrote the entire section entitled "Silver nanoparticles (AgNPs): Possible impacts on environment, health, and safety (EHS)". KM, AM, MW, and JV reviewed the manuscript text.

### Conflict of interest statement

The authors declare that the research was conducted in the absence of any commercial or financial relationships that could be construed as a potential conflict of interest.

## References

[B1] Agnihotri S. Mukherji S. Mukherji S. (2014). Size-controlled silver nanoparticles synthesized over the range 5–100 nm using the same protocol and their antibacterial efficacy. RSC Adv. 4, 3974–3983. 10.1039/c3ra44507k

[B2] Ahamed M. Posgai R. Gorey T. J. Nielsen M. Hussain S. M. Rowe J. J. (2010). Silver nanoparticles induced heat shock protein 70, oxidative stress and apoptosis in *Drosophila melanogaster*. Toxicol. Appl. Pharmacol. 242, 263–269. 10.1016/j.taap.2009.10.016 19874832

[B3] Ahmad R. Griffete N. Lamouri A. Felidj N. Chehimi M. M. Mangeney C. (2015). Nanocomposites of gold nanoparticles@molecularly imprinted polymers: chemistry, processing, and applications in sensors. Chem. Mater. 27, 5464−5478. 10.1021/acs.chemmater.5b00138

[B4] Aitken R. J. Hankin S. M. Lang Tran C. Donaldson K. Stone V. Cumpson P. (2008). A multidisciplinary approach to the identification of reference materials for engineered nanoparticle toxicology. Nanotoxicology 2, 71–78. 10.1080/17435390802109177

[B5] Alemán J. V. Chadwick A. V. He J. Hess M. Horie K. Jones R. G. (2007). Definitions of terms relating to the structure and processing of sols, gels, networks, and inorganic-organic hybrid materials (IUPAC Recommendations 2007). Pure Appl. Chem. 79, 1801–1829. 10.1351/pac200779101801

[B6] Almofti M. R. Ichikawa T. Yamashita K. Terada H. Shinohara Y. (2003). Silver ion induces a cyclosporine a-insensitive permeability transition in rat liver mitochondria and release of apoptogenic cytochrome C. J. Biochem. 134, 43–49. 10.1093/jb/mvg111 12944369

[B7] Alshehri A. H. Jakubowska M. Młozniak A. Horaczek M. Rudka D. Free C.. (2012). Enhanced electrical conductivity of silver nanoparticles for high frequency electronic applications. ACS Appl. Mater. Interfaces 4, 7007–7010. 10.1021/am3022569 23151185

[B8] Anjum N. A. Gill S. S. Duarte A. C. Pereira E. Ahmad I. (2013). Silver nanoparticles in soil–plant systems. J. Nanopart. Res. 15, 1896 10.1007/s11051-013-1896-7

[B9] Argentiere S. Cella C. Cesaria M. Milani P. Lenardi C. (2016). Silver nanoparticles in complex biological media: assessment of colloidal stability and protein corona formation. J. Nanopart. Res. 18, 253 10.1007/s11051-016-3560-5

[B10] Arora S. Jain J. Rajwade J. M. Paknikar K. M. (2008). Cellular responses induced by silver nanoparticles: *in vitro* studies. Toxicol. Lett. 179, 93–100. 10.1016/j.toxlet.2008.04.009 18508209

[B11] Asharani P. V. Hande M. P. Valiyaveettil S. (2009a). Anti-proliferative activity of silver nanoparticles. BMC cell Biol. 10:65. 10.1186/1471-2121-10-65 19761582 PMC2759918

[B12] Asharani P. V. Low Kah Mun G. Hande M. P. Valiyaveettil S. (2009b). Cytotoxicity and genotoxicity of silver nanoparticles in human cells. ACS Nano 3, 279–290. 10.1021/nn800596w 19236062

[B13] ASTM E2456-06 (2012). Standard Terminology Relating to Nanotechnology. ASTM International. Available online at: https://www.astm.org/cgi-bin/resolver.cgi?E2456-06(2012)

[B14] ASTM E2490-09 (2015). Standard Guide for Measurement of Particle Size Distribution of Nanomaterials in Suspension by Photon Correlation Spectroscopy (PCS). West Conshohocken, PA: ASTM International Available online at: http://dx.doi.org/10.1520/E2490-09R15

[B15] ASTM E2834-12 (2012). Standard Guide for Measurement of Particle Size Distribution of Nanomaterials in Suspension by Nanoparticle Tracking Analysis (NTA). ASTM International. Available online at: http://dx.doi.org/10.1520/E2834-12

[B16] ASTM E2859-11 (2011). Standard Guide for Size Measurement of Nanoparticles Using Atomic Force Microscopy. ASTM International. Available online at: http://dx.doi.org/10.1520/E2859-11

[B17] ASTM E2864-13 (2013) Standard Test Method for Measurement of Airborne Metal and Metal Oxide Nanoparticle Surface Area Concentration in Inhalation Exposure Chambers using Krypton Gas Adsorption. West Conshohocken, PA: ASTM International, 2013. Available online at: https://doi.org/10.1520/E2864

[B18] ASTM WK39049 New Guide for Sample Preparation of Charge-Stabilized Metal Nanoparticles for Electron Microscopy. (document under development). Available online at: https://www.astm.org/COMMIT/List%20of%20E56%20Standards%20and%20Work%20Items%2011-2014.doc

[B19] ASTM WK54613 New Guide for Standard Guide for the Analysis of Nanoparticles by Single Particle Inductively Coupled Plasma Mass Spectrometry (SP-ICP-MS). (document under development). Available online at: https://www.astm.org/COMMIT/SUBCOMMIT/E5602.htm

[B20] ASTM WK54872 New Test Method for Measuring the Size of Nanoparticles in Aqueous Media Using Batch-Mode Dynamic Light Scattering. (document under development). Available online at: https://www.astm.org/COMMIT/SUBCOMMIT/E5602.htm

[B21] ATSDR (1990). Toxicological Profile for Silver. TP-90-24. Atlanta, GA: Agency for Toxic Substances and Disease Registry. 38091457

[B22] Babick F. Mielke J. Wohlleben W. Weigel S. Hodoroaba V. D. (2016). How reliably can a material be classified as a nanomaterial? Available particle-sizing techniques at work. J. Nanopart. Res. 18, 1–40. 10.1007/s11051-016-3461-7 27375365 PMC4908171

[B23] Baer D. R. Gaspar D. J. Nachimuthu P. Techane S. D. Castner D. G. (2010). Application of surface chemical analysis tools for characterization of nanoparticles. Anal. Bioanal. Chem. 396, 983–1002. 10.1007/s00216-009-3360-1 20052578 PMC2841528

[B24] Bastús N. G. Merkoçi F. Piella J. Puntes V. (2014). Synthesis of highly monodisperse citrate-stabilized silver nanoparticles of up to 200 nm: kinetic control and catalytic properties. Chem. Mater. 26, 2836−2846. 10.1021/cm500316k

[B25] Bayram S. Zahr O. Blum A. M. (2015). Short ligands offer long-term water stability and plasmon tunability for silver nanoparticles. RSC Adv. 5, 6653–6559. 10.1039/C4RA09667C

[B26] Bell R. A. Kramer J. R. (1999). Structural chemistry and geochemistry of silver-sulfur compounds: critical review. Environ. Toxicol. Chem. 18, 9–22. 10.1002/etc.5620180103

[B27] Benn T. M. Westerhoff P. (2008). Nanoparticle silver released into water from commercially available sock fabrics. Environ. Sci. Technol. 42, 4133–4139. 10.1021/es7032718 18589977

[B28] BIPM I., IFCC I., IUPAC I., ISO O, (2008). The International Vocabulary of Metrology—Basic and General Concepts and Associated Terms (VIM), 3rd Edn. JCGM 200: 2012. JCGM (Paris: Joint Committee for Guides in Metrology). Available online at: http://www.bipm.org/en/publications/guides/vim.html

[B29] Birol H. Renato C. Guiotokuc M. Hotzab D. (2013). Preparation of ceramic nanoparticles via cellulose assisted glycine nitrate process: a review. RSC Adv. 3, 2873–2884. 10.1039/c2ra21810k

[B30] Blaser S. A. Scheringer M. MacLeod M. Hungerbühler K. (2008). Estimation of cumulative aquatic exposure and risk due to silver: contribution of nano-functionalized plastics and textiles. Sci. Tot. Environ. 390, 396–409. 10.1016/j.scitotenv.2007.10.010 18031795

[B31] Bollella P. Schulz C. Favero G. Mazzei F. Ludwig R. Gorton L. (2017). Green synthesis and characterization of gold and silver nanoparticles and their application for development of a third generation lactose biosensor. Electroanal 29, 77–86. 10.1002/elan.201600476

[B32] Bonevich J. E. Haller W. K. NIST–NCL Method PCC- (2010). Measuring the Size of Nanoparticles Using Transmission Electron Microscopy (TEM). Gaithersburg, MD: National Institute of Standards and Technology Available online at: https://www.nist.gov/publications 39013049

[B33] Boverhof D. R. Bramante C. M. Butala J. H. Clancy S. F. Lafranconi M. West J.. (2015). Comparative assessment of nanomaterial definitions and safety evaluation considerations. Regul. Toxicol. Pharm. 73, 137–150. 10.1016/j.yrtph.2015.06.001 26111608

[B34] Braun A. Couteau O. Franks K. Kestens V. Roebben G. Lamberty A. (2011a). Validation of dynamic light scattering and centrifugal liquid sedimentation methods for nanoparticle characterisation. Adv. Powder Technol. 22, 766–770. 10.1016/j.apt.2010.11.001

[B35] Braun A. Franks K. Kestens V. Roebben G. Lamberty A. Linsinger T. (2011b). Certification of Equivalent Spherical Diameters of Silica Nanoparticles in Water. ERM-FD100, Report EUR, 24620.

[B36] Braun A. Kestens V. Franks K. Roebben G. Lamberty A. Linsinger T. P. (2012). A new certified reference material for size analysis of nanoparticles. J. Nanopart. Res. 14, 1–12. 10.1007/s11051-012-1021-322448125

[B37] Braydich-Stolle L. K. Lucas B. Schrand A. Murdock R. C. Lee T. Schlager J. J.. (2010). Silver nanoparticles disrupt GDNF/Fyn kinase signaling in spermatogonial stem cells. Toxicol. Sci. 116, 577–589. 10.1093/toxsci/kfq148 20488942 PMC2905406

[B38] Brunauer S. Emmett P. H. Teller E. (1938). Adsorption of gases in multimolecular layers. J. Am. Chem. Soc. 60, 309–319. 10.1021/ja01269a023

[B39] Buhr E. Senftleben N. Klein T. Bergmann D. Gnieser D. Frase C. G. (2009). Characterization of nanoparticles by scanning electron microscopy in transmission mode. Meas. Sci. Technol. 20:084025 10.1088/0957-0233/20/8/084025

[B40] Burrell R. E (2003). A scientific perspective on the use of topical silver preparations. Ostomy Wound Manage. 49 (5Suppl.), 19–24. Available online at: http://www.o-wm.com/content/a-scientific-perspective-use-topical-silver-preparations 12883161

[B41] Cakić M. Glišić S. Nikolić G. Nikolić G. M. Cakić K. Cvetinov M. (2016). Synthesis, characterization and antimicrobial activity of dextran sulphate stabilized silver nanoparticles. J. Mol. Struct. 1110, 156–161. 10.1016/j.molstruc.2016.01.040

[B42] Calahorra Y. Shtempluck O. Kotchetkov V. Yaish Y. E. (2016). Young's modulus, residual stress, and crystal orientation of doubly clamped silicon nanowire beams. Nano Lett. 15, 2945–2950. 10.1021/nl5047939 25826449

[B43] Carlson C. Hussain S. M. Schrand A. M. K. Braydich-Stolle L. Hess K. L. Jones R. (2008). Unique cellular interaction of silver nanoparticles: size-dependent generation of reactive oxygen species. J. Phys. Chem. B 112, 13608–13619. 10.1021/jp712087m 18831567

[B44] Chairuangkitti P. Lawanprasert S. Roytrakul S. Aueviriyavit S. Phummiratch D. Kulthong K.. (2013). Silver nanoparticles induce toxicity in A549 cells via ROS-dependent and ROS-independent pathways. Toxicol. In Vitro 27, 330–338. 10.1016/j.tiv.2012.08.021 22940466

[B45] Chen H. Roco M. C. Li X. Lin Y. (2008a). Trends in nanotechnology patents. Nat. Nanotechnol. 3, 123–125. 10.1038/nnano.2008.51 18654475

[B46] Chen J. Wang J. Zhang X. Jin Y. (2008b). Microwave-assisted green synthesis of silver nanoparticles by carboxymethyl cellulose sodium and silver nitrate. Mater. Chem. Phys. 108, 421–424. 10.1016/j.matchemphys.2007.10.019

[B47] Chunfa D. Xianglin Z. Hao C. Chuanliang C. (2016). Sodium alginate mediated route for the synthesis of monodisperse silver nanoparticles using glucose as reducing agents. Rare Metal Mater. Eng. 45, 261–266. 10.1016/S1875-5372(16)30051-0

[B48] Cientifica (2011). Global Funding of Nanotechnologies and Its Impact. London: Cientifica Available online at: http://cientifica.com/wp-content/uploads/downloads/2011/07/Global-Nanotechnology-Funding-Report-2011.pdf

[B49] Commission Recommendation (2011). Commission Recommendation of 18 October 2011 on the definition of nanomaterial 2011/696/EU. Off. J. Eur. Union L 275, 38–40. Available online at: http://eur-lex.europa.eu/legal-content/EN/ALL/?uri=OJ%3AL%3A2011%3A275%3ATOC

[B50] Contado C. (2015). Nanomaterials in consumer products: a challenging analytical problem. Front. Chem. 3:48. 10.3389/fchem.2015.00048 26301216 PMC4527077

[B51] Cornelis G. DooletteMadeleine Thomas C. McLaughlin M. J. Kirby J. K. Beak D. G. Chittleborough D. (2012). Retention and dissolution of engineered silver nanoparticles in natural soils. Soil Sci. Soc. Am. J. 76, 891–902. 10.2136/sssaj2011.0360

[B52] Costa-Fernandez J. M. Menendez-Miranda M. Bouzas-Ramos D. Saenz-Medel A. Encinar J. R. (2016). Mass spectrometry for the characterization and quantification of engineered inorganic nanoparticles. Trends Anal Chem. TrAC 84, 139–148. 10.1016/j.trac.2016.06.001

[B53] Croce T. A. (2014). FDA's Regulation of nanotechnology in food ingredients. chemistry of food, food supplements, and food contact materials: from production to plate. Am. Chem. Soc. Chapter 4, 41–50. 10.1021/bk-2014-1159.ch004

[B54] Cunningham A. Bürgi T. (2013). Chapter 1 Bottom-up Organisation of Metallic” Nanoparticles,, in Amorpphous Nanophotonics, eds Rockstuhl C. Scharf T. (Berlin; Heidelberg: Springer).

[B55] Cushing B. L. Kolesnichenko V. L. O'Connor C. J. (2014). Recent advances in the liquid-phase syntheses of inorganic nanoparticles. Chem. Rev. 104, 3893–3946. 10.1021/cr030027b 15352782

[B56] Dadosh T. (2009). Synthesis of uniform silver nanoparticle with a controllable size. Mater. Lett. 63, 2236–2238. 10.1016/j.matlet.2009.07.042

[B57] Darroudi M. Bin Ahmad M. B. Abdullah A. H. Ibrahim N. A. (2011). Green synthesis and characterization of gelatin-based and sugar-reduced silver nanoparticles. Int. J. Nanomedicine 6, 569–574. 10.2147/ijn.s16867 21674013 PMC3107715

[B58] De Temmerman P. J. Lammertyn J. De Ketelaere B. Kestens V. Roebben G. Verleysen E. (2014). Measurement uncertainties of size, shape, and surface measurements using transmission electron microscopy of near-monodisperse, near-spherical nanoparticles. J. Nanopart. Res. 16, 1–22. 10.1007/s11051-013-2177-1

[B59] Decree No 2012-232 Annual Declaration on Substances at Nanoscale in Application of Article R. 523-4 of the Environment Code. France: Official journal of the French Republic Available online at: https://www.r-nano.fr/?locale=en

[B60] Decree No 2014/24329 Royal Decree on the Placing on the Market of Substances Manufactured with Nanoparticle Status. Belgium: Belgian Official Journal Available online at: http://www.health.belgium.be/fr/environnement/substances-chimiques/nanomateriaux/l-registre

[B61] Decree No 644 of 13/06/2014 On a Register of Mixtures Articles that Contain Nanomaterials as well as the Requirement for Producers Importers to Report to the Register. Danmark.

[B62] Delvallée A. Feltin N. Ducourtieux S. Trabelsi M. Hochepied J. F. (2015a). Toward an uncertainty budget for measuring nanoparticles by AFM. Metrologia 53:41 10.1088/0026-1394/53/1/41

[B63] Delvallée A. Feltin N. Ducourtieux S. Trabelsi M. Hochepied J. F. (2015b). Direct comparison of AFM and SEM measurements on the same set of nanoparticles. Meas. Sci. Technol. 26:085601 10.1088/0957-0233/26/8/085601

[B64] DiVincenzo G. D. Giordano C. J. Schriever L. S. (1985). Biologic monitoring of workers exposed to silver. Int. Arch. Occup. Environ. Health 56, 207–215. 10.1007/BF00396598 4066049

[B65] Dong X. Ji X. Wu H. Zhao L. Li J. Yang W. (2009). Shape control of silver nanoparticles by stepwise citrate reduction. J. Phys. Chem. C 113, 6573–6576. 10.1021/jp900775b

[B66] Drake P. L. Hazelwood K. J. (2005). Exposure-related health effects of silver and silver compounds: a review. Ann. Occup. Hyg. 49, 575–585. 10.1093/annhyg/mei019 15964881

[B67] Dudkiewicz A. Boxall A. B. Chaudhry Q. Mølhave K. Tiede K. Hofmann P.. (2015). Uncertainties of size measurements in electron microscopy characterization of nanomaterials in foods. Food Chem. 176, 472–479. 10.1016/j.foodchem.2014.12.071 25624258

[B68] Ediriwickrema A. Saltzman W. M. (2015). Nanotherapy for Cancer: targeting and multifunctionality in the future of cancer therapies. ACS Biomater. Sci. Eng. 1, 64−78. 10.1021/ab500084g 25984571 PMC4426346

[B69] Elsupikhe R. F. Shameli K. Ahmad M. B. Ibrahim N. A. Zainudin N. (2015). Green sonochemical synthesis of silver nanoparticles at varying concentrations of κ-carrageenan. Nanoscale Res. Lett. 10:1. 10.1186/s11671-015-0916-1 26220106 PMC4523502

[B70] Environmental Protection Agency (2015). (EPA), Chemical substances when manufactured or processed as nanoscale materials: TSCA reporting and recordkeeping requirements Fed. Regist. 80 18330 Available online at: http://www.regulations.gov

[B71] Fabrega J. Luoma S. N. Tyler C. R. Galloway T. S. Lead J. R. (2011). Silver nanoparticles: behaviour and effects in the aquatic environment. Environ. Int. 37, 517–531. 10.1016/j.envint.2010.10.012 21159383

[B72] Fabricius A. L. Duester L. Meermann B. Ternes T. A. (2014). ICP-MS-based characterization of inorganic nanoparticles—sample preparation and off-line fractionation strategies. Anal. Bioanal. Chem. 406, 467–479. 10.1007/s00216-013-7480-2 24292431 PMC3885803

[B73] Finney E. E. Finke R. G. (2008). Nanocluster nucleation and growth kinetic and mechanistic studies: a review emphasizing transition-metal nanoclusters. J. Colloid Interface Sci. 317, 351–374. 10.1016/j.jcis.2007.05.092 18028940

[B74] Fiorino D. (2010). Voluntary Initiatives, Regulation and Nanotechnology Oversight. Project on Emerging Nanotechnologies is Supported, Woodrow Wilson International Center for Scholar, 2010. Available online at: https://www.wilsoncenter.org/publication/pen-19-voluntary-initiatives-regulation-and-nanotechnology-oversight

[B75] Foldbjerg R. Jiang X. Miclăuş T. Chen C. Autrup H. Beer C. (2015). Silver nanoparticles–wolves in sheep's clothing?. Toxicol. Res. 4, 563–575. 10.1039/C4TX00110A

[B76] Franks K. Braun A. Charoud-Got J. Couteau O. Kestens V. Lamberty A. (2012). Certification of the Equivalent Spherical Diameters of Silica Nanoparticles in Aqueous Solution. ERM-FD304, Report EUR, 25018.

[B77] Gallego-Urrea J. A. Tuoriniemi J. Hassellöv M. (2011). Applications of particle-tracking analysis to the determination of size distributions and concentrations of nanoparticles in environmental, biological and food samples. TrAC-Trends Anal. Chem. 30, 473–483. 10.1016/j.trac.2011.01.005

[B78] Gawande M. B. Goswami A. Felpin F. X. Asefa T. Huang X. Silva R.. (2016). Cu and Cu-based nanoparticles: synthesis and applications in catalysis. Chem. Rev. 116, 3722–3811. 10.1021/acs.chemrev.5b00482 26935812

[B79] Geranio L. Heuberger M. Nowack B. (2009). The behavior of silver nanotextiles during washing. Environ. Sci. Technol. 43, 8113–8118. 10.1021/es9018332 19924931

[B80] Gliga A. R. Skoglund S. Wallinder I. O. Fadeel B. Karlsson H. L. (2014). Size-dependent cytotoxicity of silver nanoparticles in human lung cells: the role of cellular uptake, agglomeration and Ag release. Part Fibre Toxicol. 11:1. 10.1186/1743-8977-11-11 24529161 PMC3933429

[B81] Gomes S. I. Hansen D. Scott-Fordsmand J. J. Amorim M. J. (2015). Effects of silver nanoparticles to soil invertebrates: oxidative stress biomarkers in *Eisenia fetida*. Environ. Pollut. 199, 49–55. 10.1016/j.envpol.2015.01.01 25618366

[B82] Gomes S. I. Soares A. M. Scott-Fordsmand J. J. Amorim M. J. (2013). Mechanisms of response to silver nanoparticles on *Enchytraeus albidus* (Oligochaeta): survival, reproduction and gene expression profile. J. Hazard Mater. 254, 336–344. 10.1016/j.jhazmat.2013.04.005 23644687

[B83] Gorham J. M. Rohlfing A. B. Lippa K. A. MacCuspie R. I. Hemmati A. Holbrook R. D. (2014). Storage Wars: how citrate-capped silver nanoparticle suspensions are affected by not-so-trivial decisions. J. Nanopart. Res. 16, 1–14. 10.1007/s11051-014-2339-9

[B84] Gorham J. MacCuspie R. I. Klein K. L. Fairbrother H. D. Holbrook R. D. (2012). Uv-induced photochemical transformation of citrate-capped silver nanoparticle suspension. J. Nanopart. Res. 14, 1139 10.1007/s11051-012-1139-3

[B85] Gottschalk F. Nowack B. (2011). The release of engineered nanomaterials to the environment. J. Environ. Monit. 13, 1145–1155. 10.1039/C0EM00547A 21387066

[B86] Gottschalk F. Sonderer T. Scholz R. W. Nowack B. (2009). Modeled environmental concentrations of engineered nanomaterials (TiO2, ZnO, Ag, CNT, fullerenes) for different regions. Environ. Sci. Technol. 43, 9216–9222. 10.1021/es9015553 20000512

[B87] Grobelny J. Delrio F. W. Pradeep N. Kim D. I. Hackley V. A. Cook R. F (2009). NIST—NCL Joint Assay Protocol, PCC-6: Size Measurement of Nanoparticles Using Atomic Force Microscopy. Gaithersburg, MD: NIST Available online at: https://www.nist.gov/publications

[B88] Grombe R. Allmaier G. Charoud-Got J. Dudkiewicz A. Emteborg H. Hofmann T. (2015). Feasibility of the development of reference materials for the detection of Ag nanoparticles in food: neat dispersions and spiked chicken meat. Accred. Qual. Assur. 20, 3–16. 10.1007/s00769-014-1100-5

[B89] Gunsolus I. L. Haynes C. L. (2015). Analytical aspects of nanotoxicology. Anal. Chem. 88, 451–479. 10.1021/acs.analchem.5b04221 26565109

[B90] Hackley V. A. Clogston J. D. (2015). NIST Special Publication 1200-6 (2015). Measuring the Size of Nanoparticles in Aqueous Media Using Batch-Mode Dynamic Light Scattering. Gaithersburg, MD: National Institute of Standards and Technology Available online at: https://www.nist.gov/publications

[B91] Hamburg M. A. (2012). FDA's approach to regulation of products of nanotechnology. Science 336, 299–300. 10.1126/science.1205441 22517845

[B92] Hänsch M. Emmerling C. (2010). Effects of silver nanoparticles on the microbiota and enzyme activity in soil. J. Plant Nutr. Soil Sci. 173, 554–558. 10.1002/jpln.200900358

[B93] Hansen S. F. Larsen B. H. Olsen S. I. Baun A. (2007). Categorization framework to aid hazard identification of nanomaterials. Nanotoxicology 1, 243–253. 10.1080/17435390701727509

[B94] Hartmann G. Hutterer C. Schuster M. (2013). Ultra-trace determination of silver nanoparticles in water samples using cloud point extraction and ETAAS. J Anal. Atom. Spectrom. 28, 567–572. 10.1039/C3JA30365A

[B95] Hayashi Y. Engelmann P. Foldbjerg R. Szabó M. Somogyi I. Pollák E.. (2012). Earthworms and humans *in vitro*: characterizing evolutionarily conserved stress and immune responses to silver nanoparticles. Environ. Sci. Technol. 46, 4166–4173. 10.1021/es3000905 22432789

[B96] Hayashi Y. Heckmann L. H. Simonsen V. Scott-Fordsmand J. J. (2013). Time-course profiling of molecular stress responses to silver nanoparticles in the earthworm *Eisenia fetida*. Ecotoxicol. Environ. Saf. 98, 219–226. 10.1016/j.ecoenv.2013.08.017 24041528

[B97] Health Canada (2011). Health Canada, Policy Statement on Health Canada's Working Definition for Nanomaterial. (2011) Available online at: http://www.hc-sc.gc.ca/sr-sr/pubs/nano/pol-eng.php

[B98] Hendren C. O. Mesnard X. Dröge J. Wiesner M. R. (2011). Estimating production data for five engineered nanomaterials as a basis for exposure assessment. Environ. Sci. Technol. 45, 2562–2569. 10.1021/es103300g 21391627

[B99] Hill W. R. (1941). Argyria The pharmacology of silver. South Med. J. 34, 340.

[B100] Hodoroaba V. D. Rades S. Salge T. Mielke J. Ortel E. Schmidt R. (2016). Characterisation of nanoparticles by means of high-resolution SEM/EDS in transmission mode,, in IOP Conference Series: Materials Science and Engineering, Vol. 109 (Portoroz: IOP Publishing). 10.1088/1757-899X/109/1/012006

[B101] Hofmann M. Grainger D. W. Hofmann H. (2015). Nanoparticles in medicine: current challenges facing inorganic nanoparticle toxicity assessments and standardizations. Nanomed. Nanotechnol. 11 1689–1694. 10.1016/j.nano.2015.05.005 26051651

[B102] Hohenester U. Ditlbacher H. Krenn J. R. (2009). Electron-energy-loss spectra of plasmonic nanoparticles. Phy. Rev. Lett. 103:106801. 10.1103/PhysRevLett.103.106801 19792333

[B103] Hole P. Sillence K. Hannell C. Maguire C. M. Roesslein M. Suarez G.. (2013). Interlaboratory comparison of size measurements on nanoparticles using nanoparticle tracking analysis (NTA). J. Nanopart. Res. 15, 1–12. 10.1007/s11051-013-2101-8 24348090 PMC3857864

[B104] Hollinger M. A. (1996). Toxicological aspects of topical silver pharmaceuticals. Crit. Rev. Toxicol. 26, 255–260. 10.3109/10408449609012524 8726163

[B105] Hoo C. M. Starostin N. West P. Mecartney M. L. (2008). A comparison of atomic force microscopy (AFM) and dynamic light scattering (DLS) methods to characterize nanoparticle size distributions. J. Nanopart. Res. 10, 89–96. 10.1007/s11051-008-9435-7

[B106] Hornyak G. L. Dutta J. Tibbals H. F. Miller F. P. (2008). Introduction to Nanosciences. Boca Raton, FL: CRC Press, Taylor and Francis Group.

[B107] ISO 13318-1 (2001). Determination of Particle Size Distribution by Centrifugal Liquid Sedimentation Methods – Part 1: General Principles and Guidelines. Geneva: International Organization for Standardization.

[B108] ISO 13322-1 (2014). Particle Size Analysis - Image Analysis Methods - Part 1: Static Image Analysis Methods. Geneva: International Organization for Standardization.

[B109] ISO 15900 (2009). Determination of Particle Size Distribution - Differential Electrical Mobility Analysis for Aerosol Particles. Geneva: International Organization for Standardization.

[B110] ISO 17867 (2015). Particle Size Analysis - Small-Angle X-ray Scattering. Geneva: International Organization for Standardization.

[B111] ISO 20998-2 (2013). Measurement and Characterization of Particles by Acoustic Methods — Part 2: Guidelines for Linear Theory. Geneva: International Organization for Standardization.

[B112] ISO 22412 (2008). Particle Size Analysis - Dynamic Light Scattering (DLS). Geneva: International Organization for Standardization.

[B113] ISO 29301 (2010). Microbeam Analysis - Analytical Transmission Electron Microscopy - Methods for Calibrating Image Magnification by Using Reference Materials having Periodic Structures. Geneva: International Organization for Standardization.

[B114] ISO 9277 (2010). Determination of the specific surface area of solids by gas adsorption - BET method. Geneva: International Organization for Standardization.

[B115] ISO 19430 (2016). Particle Size Analysis - Particle Tracking Analysis (PTA) Method. Geneva: International Organization for Standardization. (document under development).

[B116] ISO/Guide 30 Reference Materials - Selected Terms Definitions. Geneva: International Organization for Standardization.

[B117] ISO/Guide 34 (2009). General Requirements for the Competence of Reference Material Producers. Geneva: International Organization for Standardization.

[B118] ISO/IEC Guide 98-3 (2008). Uncertainty of Measurement - Part 3: Guide to the Expression of Uncertainty in Measurement (GUM:1995). Geneva: International Organization for Standardization.

[B119] ISO/TS 1410 (2012). Surface Characterization of Gold Nanoparticles for Nanomaterial Specific Toxicity Screening: FT-IR Method. Geneva: International Organization for Standardization.

[B120] ISO/TS 16195 (2013). Guidance for Developing Representative Test Materials Consisting of Nano-objects in Dry Powder Form. Geneva: International Organization for Standardization.

[B121] ISO/TS 19590. Nanotechnologies - Size Distribution Concentration of Inorganic Nanoparticles in Aqueous Media via Single Particle Inductively Coupled Plasma Mass Spectrometry. Geneva: International Organization for Standardization. (document under development)

[B122] ISO/TS 80004-1 (2015). Nanotechnologies-Vocabulary-Part 1: Core Terms. Geneva: International Organization for Standardization.

[B123] ISO/TS 80004-2 (2015). Nanotechnologies-Vocabulary-Part 2: Nano-Objects. Geneva: International Organization for Standardization.

[B124] ISO/TS 80004-4 (2011). Nanotechnologies-Vocabulary-Part 4: Nanostructured Materials. Geneva: International Organization for Standardization.

[B125] Jana N. R. Gearheart L. Murphy C. J. (2001). Evidence for seed-mediated nucleation in the chemical reduction of gold salts to gold nanoparticles. Chem. Mater. 13, 2313–2322. 10.1021/cm000662n

[B126] Jena P. Mohanty S. Mallick R. Jacob B. Sonawane A. (2012). Toxicity and antibacterial assessment of chitosan-coated silver nanoparticles on human pathogens and macrophage cells. Int. J. Nanomedicine 7, 1805–1818. 10.2147/IJN.S28077 22619529 PMC3356211

[B127] Jiang L. P. Wang A. N. Zhao Y. Zhang J. R. Zhu J. J. (2004). A novel route for the preparation of monodisperse silver nanoparticles via a pulsed sonoelectrochemical technique, Inorg. Chem. Commun. 7, 506–509. 10.1016/j.inoche.2004.02.003

[B128] Jiang X. Foldbjerg R. Miclaus T. Wang L. Singh R. Hayashi Y.. (2013). Multi-platform genotoxicity analysis of silver nanoparticles in the model cell line CHO-K1. Toxicol. Lett. 222, 55–63. 10.1016/j.toxlet.2013.07.011 23872614

[B129] Jo Y. K. Kim B. H. Jung G. (2009). Antifungal activity of silver ions and nanoparticles on phytopathogenic fungi. Plant Dis. 93, 1037–1043. 10.1094/PDIS-93-10-1037 30754381

[B130] Joint Research Center (2016). Nanomaterial Repository. Available online at: https://ec.europa.eu/jrc/en/scientific-tool/jrc-nanomaterials-repository (Accessed September 13, 2016).

[B131] Jung J. H. Kim S. W. Min J. S. Kim Y. J. Lamsal K. Kim K. S.. (2010). The effect of nano-silver liquid against the white rot of the green onion caused by Sclerotium cepivorum. Mycobiology 38, 39–45. 10.4489/MYCO.2010.38.1.039 23956623 PMC3741593

[B132] Kang I. K. Haider A. (2015). Preparation of silver nanoparticles and their industrial and biomedical applications: a comprehensive review. Adv. Mater. Sci. 2015, 16 10.1155/2015/165257

[B133] Keat C. L. Aziz A. Eid A. M. Elmarzugi N. A. (2015). Biosynthesis of nanoparticles and silver nanoparticles. Biorerour. Bioprocess. 2:47 10.1186/s40643-015-0076-2

[B134] Keller A. A. McFerran S. Lazareva A. Suh S. (2013). Global life cycle releases of engineered nanomaterials J. Nanopart. Res. 15, 1692 10.1007/s11051-013-1692-4

[B135] Kelly K. L. Coronado E. Zhao L. L. Schatz G. C. (2003). The optical properties of metal nanoparticles: the influence of size, shape, and dielectric environment. J. Phys. Chem. B. 107, 668–677. 10.1021/jp026731y

[B136] Kemp M. M. Kumar A. Clement D. Ajayan P. Mousa S. Linhardt R. J. (2009). Hyaluronan-and heparin-reduced silver nanoparticles with antimicrobial properties. Nanomedicine 4, 421–429. 10.2217/nnm.09.24 19505245 PMC2717895

[B137] Kestens V. Roebben G. Herrmann J. Jämting Å. Coleman V. Minelli C.. (2016). Challenges in the size analysis of a silica nanoparticle mixture as candidate certified reference material. J. Nanopart. Res. 18, 1–22. 10.1007/s11051-016-3474-2 27441027 PMC4917587

[B138] Kettemann F. Birnbaum A. Witte S. Wuithschick M. Pinna N. Kraehnert R. (2016). Missing piece of the mechanism of the turkevich method: the critical role of citrate protonation. Chem. Mater. 28, 4072–4081. 10.1021/acs.chemmater.6b01796

[B139] Kim B. Park C. S. Murayama M. Hochella M. F. Jr. (2010). Discovery and characterization of silver sulfide nanoparticles in final sewage sludge products. Environ. Sci. Technol. 44, 7509–7514. 10.1021/es101565j 20839838

[B140] Kim D. Jeong S. Moon J. (2006). Synthesis of silver nanoparticles using the polyol process and the influence of precursor injection. Nanotechnology 17, 4019–4024. 10.1088/0957-4484/17/16/004 21727531

[B141] Kim S. W. Kim K. S. Lamsal K. Kim Y. J. Kim S. B. Jung M.. (2009b). An in vitro study of the antifungal effect of silver nanoparticles on oak wilt pathogen Raffaelea sp. J Micro. Biotechnol. 19, 760–764. 10.4014/jmb.0812.649 19734712

[B142] Kim S. Ryu D. Y. (2013). Silver nanoparticle-induced oxidative stress, genotoxicity and apoptosis in cultured cells and animal tissues. J. Appl. Toxicol. 33, 78–89. 10.1002/jat.2792 22936301

[B143] Kim S. Choi J. E. Choi J. Chung K. H. Park K. Yi J.. (2009a). Oxidative stress-dependent toxicity of silver nanoparticles in human hepatoma cells. Toxicol. In Vitro 23, 1076–1084. 10.1016/j.tiv.2009.06.001 19508889

[B144] Kim Y. P. Shon H. K. Shin S. K. Lee T. G. (2015). Probing nanoparticles and nanoparticle-conjugated biomolecules using time-of-flight secondary ion mass spectrometry. Mass Spectrom. Rev. 34, 237–247. 10.1002/mas.21437 24890130

[B145] Klabunde K. J. (2001). Nanoscale Materials in Chemistry. New York, NY: John Wiley & Sons, Inc.

[B146] Klein C. L. Comero S. Stahlmecke B. Romazanov J. Kuhlbusch T. A. J. Van Doren E. (2011a). NM-Series of Representative Manufactured Nanomaterials, NM-300 Silver, Characterisation, Stability, Homogeneity. EUR 24693 EN. ISBN 978-92-79-19068-1

[B147] Klein T. Buhr E. Johnsen K. P. Frase C. G. (2011b). Traceable measurement of nanoparticle size using a scanning electron microscope in transmission mode (TSEM). Meas. Sci. Technol. 22:094002 10.1088/0957-0233/22/9/094002

[B148] Korani M. Ghazizadeh E. Korani S. Hami Z. Mohammadi-Bardbori A. (2015). Effects of silver nanoparticles on human health. Eur. J. Nanomed. 7, 51–62. 10.1515/ejnm-2014-0032

[B149] Kraynov A. Müller T. E. (2011). Concepts for the Stabilization of Metal Nanoparticles in Ionic Liquids, Applications of Ionic Liquids in Science and Technology. Ed Scott Handy, InTech. Available online at: http://www.intechopen.com/books/applications-of-ionic-liquids-in-science-and-technology/concepts-for-the-stabilization-of-metal-nanoparticles-in-ionic-liquids

[B150] Kulthong K. Srisung S. Boonpavanitchakul K. Kangwansupamonkon W. Maniratanachote R. (2010). Determination of silver nanoparticle release from antibacterial fabrics into artificial sweat. Part Fibre Toxicol. 7, 8. 10.1186/1743-8977-7-8 20359338 PMC2861638

[B151] Kwon S. Y. Kim Y. G. Lee S. H. Moon J. H. (2011). Uncertainty analysis of measurements of the size of nanoparticles in aqueous solutions using dynamic light scattering. Metrologia 48, 417 10.1088/0026-1394/48/5/024

[B152] Laborda F. Bolea E. Cepriá G. Gómez M. T. Jiménez M. S. Pérez-Arantegui J.. (2016). Detection, characterization and quantification of inorganic engineered nanomaterials: a review of techniques and methodological approaches for the analysis of complex samples. Anal. Chim. Acta 904, 10–32. 10.1016/j.aca.2015.11.008 26724760

[B153] Lamsal K. Kim S. W. Jung J. H. Kim Y. S. Kim K. S. Lee Y. S. (2011a). Inhibition effects of silver nanoparticles against powdery mildews on cucumber and pumpkin. Mycobiology 39, 26–32. 10.4489/MYCO.2011.39.1.026 22783069 PMC3385079

[B154] Lamsal K. Kim S. W. Jung J. H. Kim Y. S. Kim K. S. Lee Y. S. (2011b). Application of silver nanoparticles for the control of Colletotrichum species in vitro and pepper anthracnose disease in field. Mycobiology 39, 194–199. 10.5941/MYCO.2011.39.3.194 22783103 PMC3385110

[B155] Lansdown A. (2006). Silver in health care: antimicrobial effects and safety in use,, in Biofunctional Textiles and the Skin, Vol. 33, eds Hipler U. C. Elsner P. (London: Karger Publishers), 17–34. 10.1159/000093928 16766878

[B156] Lansdown A. B. (2010). A pharmacological and toxicological profile of silver as an antimicrobial agent in medical devices. Adv. Pharmacol. Sci. 2010, 1–16. 10.1155/2010/910686 21188244 PMC3003978

[B157] Le Ouay B. Stellacci F. (2015). Antibacterial activity of silver nanoparticles: a surface science insight. Nano Today 10, 339–354. 10.1016/j.nantod.2015.04.002

[B158] Lead J. R. Tejamaya M. Römer I. Merrifield R. C. (2014). Stability of citrate, PVP, and PEG coated silver nanoparticles in ecotoxicology media. Environ. Sci. Technol. 46, 7011–7017. 10.1021/es2038596 22432856

[B159] Lee H. You S. Pikhitsa P. V. Kim J. Kwon S. Woo C. G.. (2011). Three-dimensional assembly of nanoparticles from charged aerosols. Nano Lett. 11, 119–124. 10.1021/nl103787k 21090775

[B160] Lee P. C. Meisel D. (1982). Adsorption and surface-enhanced raman of dyes on silver and gold sols. J. Phys. Chem. 86, 3391–3395. 10.1021/j100214a025

[B161] León-Silva S. Fernández-Luqueño F. López-Valdez F. (2016). Silver Nanoparticles (AgNP) in the environment: a review of potential risks on human and environmental health. Water Air Soil Pollut. 227, 306 10.1007/s11270-016-3022-9

[B162] Li T. Senesi A. J. Lee B. (2016). Small angle x-ray scattering for nanoparticle research. Chem. Rev. 116, 11128–11180. 10.1021/acs.chemrev.5b00690 27054962

[B163] Lidén G. (2011). The European commission tries to define nanomaterials. Ann. Occup. Hyg. 55, 1–5. 10.1093/annhyg/meq092 21233257

[B164] Linsinger T. P. Roebben G. Solans C. Ramsch R. (2011). Reference materials for measuring the size of nanoparticles. TrAC Trends Anal. Chem. 30, 18–27. 10.1016/j.trac.2010.09.005

[B165] Liu C. W. Chang H. W. Sarkar B. Saillard J. Y. Kahlal S. Wu Y. Y. (2009). Stable Silver (I) hydride complexes supported by diselenophosphate ligands. Inorg. Chem. 49, 468–475. 10.1021/ic901408n 20025253

[B166] Liu J. Hurt R. H. (2010). Ion release kinetics and particle persistence in aqueous nano-silver colloids. Environ. Sci. Technol. 44, 2169–2175. 10.1021/es9035557 20175529

[B167] Liu Y. Laks P. Heiden P. (2002). Controlled release of biocides in solid wood. III. Preparation and characterization of surfactant-free nanoparticles. J. Appl. Polym. Sci. 86, 615–621. 10.1002/app.10898

[B168] Lohse S. E. Murphy C. F. (2012). Applications of colloidal inorganic nanoparticles: from medicine to energy. J. Am. Chem. Soc. 134, 15607−15620. 10.1021/ja307589n 22934680

[B169] López-Lorente Á. I. Mizaikoff B. (2016). Recent advances on the characterization of nanoparticles using infrared spectroscopy. TrAC Trends Anal. Chem. 84, 97–106. 10.1016/j.trac.2016.01.012

[B170] López-Lorente A. L. Valcárcel M. (2016). The third way in analytical nanoscience and nanotechnology: involvement of nanotools and nanoanalytes in the same analytical process. Trend Anal. Chem. 9, 1–9. 10.1016/j.trac.2015.06.011

[B171] Lövestam G. Rauscher H. Roebben G. Klüttgen B. S. Gibson N. Putaud J. P. (2010). Considerations on a definition of nanomaterial for regulatory purposes. Joint Res. Centre Refer. Rep. 1, 80001–80004. 10.2788/98686

[B172] Loza K. Diendorf J. Sengstock C. Ruiz-Gonzalez L. Gonzalez-Calbet J. M. Vallet-Regi M. (2014). The dissolution and biological effects of silver nanoparticles in biological media. J. Mater. Chem. B 2, 1634–1643. 10.1039/C3TB21569E 32261391

[B173] Luther E. M. Koehler Y. Diendorf J. Epple M. Dringen R. (2011). Accumulation of silver nanoparticles by cultured primary brain astrocytes. Nanotechnology 22:375101. 10.1088/0957-4484/22/37/375101 21852719

[B174] Lux Research (2008). Lux Research. Nanomaterials State of the Market Q3 2008. Available online at: www.luxresearch.com.

[B175] MacCuspie R. I. (2011). Colloidal stability of silver nanoparticles in biologically relevant conditions. J. Nanopart. Res. 13, 2893–2908. 10.1007/s11051-010-0178-x

[B176] MacCuspie R. I. Allen A. J. Martin M. N. Hackley V. A. (2013). Just add water: reproducible singly dispersed silver nanoparticle suspensions on-demand. J. Nanopart. Res. 15, 1–12. 10.1007/s11051-013-1760-9

[B177] MacCuspie R. I. Rogers K. Patra M. Suo Z. Allen A. J. Martin M. N.. (2011). Challenges for physical characterization of silver nanoparticles under pristine and environmentally relevant conditions. J. Environ. Monit. 13, 1212–1226. 10.1039/C1EM10024F 21416095

[B178] Mader B. T. Ellefson M. E. Wolf S. T. (2015). Measurements of nanomaterials in environmentally relevant water matrices using liquid nebulization/differential mobility analysis. Environ. Toxicol. Chem. 34, 833–842. 10.1002/etc.2865 25556642

[B179] Mahmoudi M. Monopoli M. P. Rezaei M. Lynch I. Bertoli F. McManus J. J.. (2013). The protein corona mediates the impact of nanomaterials and slows amyloid beta fibrillation. ChemBioChem 14, 568–572. 10.1002/cbic.201300007 23420562

[B180] Majdalawieh A. Kanan M. C. El-Kadri O. Kanan S. M.. (2014). Recent Advances in Gold and Silver Nanoparticles: Synthesis and Applications. J. Nanosci. Nanotechnol. 14, 4757–4780. 24757945 10.1166/jnn.2014.9526

[B181] Majedy S. Y. Lee H. K. (2016). Recent advances in the separation and quantification of metallic nanoparticles and ions in the environment. TrAC Trends Anal. Chem. 75, 183–196. 10.1016/j.trac.2015.08.009

[B182] Manojkumar K. Sivaramakrishna A. Vijayakrishna K. (2016). A short review on stable metal nanoparticles using ionic liquids, supported ionic liquids, and poly(ionic liquids). J. Nanopart. Res. 18, 103 10.1007/s11051-016-3409-y

[B183] Marambio-Jones C. Hoek E. M. (2010). A review of the antibacterial effects of silver nanomaterials and potential implications for human health and the environment. J. Nanopart. Res, 12, 1531–1551. 10.1007/s11051-010-9900-y

[B184] Marbella L. E. Millstone J. E. (2015). NMR techniques for noble metal nanoparticles. Chem. Mater. 27, 2721–2739. 10.1021/cm504809c

[B185] Martin M. N. Allen A. J. MacCuspie R. I. Hackley V. A. (2014). Dissolution, agglomerate morphology, and stability limits of protein-coated silver nanoparticles. Langmuir 30, 11442–11452. 10.1021/la502973z 25137213

[B186] Maurer L. L. Meyer J. N. (2016). A systematic review of evidence for silver nanoparticle-induced mitochondrial toxicity. Environ. Sci. Nano 3, 311–322. 10.1039/c5en00187k

[B187] Mayer A. B. R. (2001). Colloidal metal nanoparticles dispersed in amphiphilic polymers. Polym. Adv. Technol. 12, 96–106. 10.1002/1099-1581(200101/02)12:1/2<96::AID-PAT943>3.0.CO;2-G

[B188] Meli F. Klein T. Buhr E. Frase C. G. Gleber G. Krumrey M. (2012). Traceable size determination of nanoparticles, a comparison among European metrology institutes. Meas. Sci. Technol. 23:125005 10.1088/0957-0233/23/12/125005

[B189] Menzel M. Bienert R. Bremser W. Girod M. Rolf S. Thünemann A. F. (2013). Certification Report Certified Reference Material BAM-N001 Particle Size Parameters of Nano Silver. Berlin: Federal Institute for Materials Research and Testing.

[B190] Meyer J. N. Lord C. A. Yang X. Y. Turner E. A. Badireddy A. R. Marinakos S. M.. (2010). Intracellular uptake and associated toxicity of silver nanoparticles in *Caenorhabditis elegans*. Aquat. Toxicol. 100, 140–150. 10.1016/j.aquatox.2010.07.016 20708279

[B191] Miller G. Wickson F. (2015). Risk analysis of nanomaterials: exposing nanotechnology's naked emperor. Rev. Policy Res. 32, 485–512. 10.1111/ropr.12129

[B192] Mitrano D. M. Rimmele E. Wichser A. Erni R. Height M. Nowack B. (2014). Presence of nanoparticles in wash water from conventional silver and nano-silver textiles. ACSnano 8, 7208–7219. 10.1021/nn502228w 24941455

[B193] Montaño M. D. Olesik J. W. Barber A. G. Challis K. Ranville J. F. (2016). Single Particle ICP-MS: advances toward routine analysis of nanomaterials. Anal. Bioanal. Chem. 408, 5053–5074. 10.1007/s00216-016-9676-8 27334719

[B194] Montoro Bustos A. R. Petersen E. J. Possolo A. Winchester M. R. (2015). Post hoc interlaboratory comparison of single particle ICP-MS size measurements of NIST gold nanoparticle reference materials. Anal. Chem. 87, 8809–8817. 10.1021/acs.analchem.5b01741 26265147

[B195] Mueller N. C. Nowack B. (2008). Exposure modeling of engineered nanoparticles in the environment. Environ. Sci. Technol. 42, 4447–4453. 10.1021/es7029637 18605569

[B196] Murphy C. J. Jana N. R. Gearheart L. (2001). Evidence for seed-mediated nucleation in the chemical reduction of gold salts to gold nanoparticles. Chem. Mater. 13, 2313–2322. 10.1021/cm000662n

[B197] Murphy K. E. Liu J. Bustos A. R. M. Johnson M. E. Winchester M. R. (2015). Characterization of Nanoparticle Suspensions Using Single Particle Inductively Coupled Plasma Mass Spectrometry. Gaithersburg, MD: NIST Special Publication.

[B198] Nath S. Jana S. Pradhan M. Pal T. (2010). Ligand-stabilized metal nanoparticles in organic solvent. J. Colloid Interfaces Sci. 341:333–352. 10.1016/j.jcis.2009.09.049 19880134

[B199] National Institute of Standards Technology NIST (2012). Certificate of Analysis, Standard Reference Material 1898, Titanium Dioxide Nanomaterial. Available online at: https://www-s.nist.gov/srmors/certificates/1898.pdf

[B200] National Institute of Standards Technology NIST (2014a). Certificate of Analysis, Standard Reference Material 1964, Nominal 60 nm Diameter Polystyrene Spheres. Available online at: https://www-s.nist.gov/srmors/certificates/1964.pdf

[B201] National Institute of Standards Technology NIST (2014b). Certificate of Analysis, Standard Reference Material 1963a, Nominal 100 nm Diameter Polystyrene Spheres. Available online at: (https://www-s.nist.gov/srmors/certificates/1963a.pdf) accessed 09.13.16.

[B202] National Institute of Standards Technology NIST (2015a). Reports of Investigation, Reference Material 8011, Gold Nanoparticles, Nominal 10 nm Diameter. Available online at: https://www-s.nist.gov/srmors/reports/8011.pdf

[B203] National Institute of Standards Technology NIST (2015b). Reports of Investigation, Reference Material 8012, Gold Nanoparticles, Nominal 30 nm Diameter. Available online at: https://www-s.nist.gov/srmors/reports/8012.pdf

[B204] National Institute of Standards Technology NIST (2015c). Reports of Investigation, Reference Material 8013, Gold Nanoparticles, Nominal 60 nm Diameter. Available online at: https://www-s.nist.gov/srmors/reports/8013.pdf

[B205] National Institute of Standards Technology NIST (2015d). Reports of Investigation, Reference Material 8017, Polyvinylpyrrolidone Coated Silver Nanoparticles, Nominal Diameter 75 nm. Available online at: https://www-s.nist.gov/srmors/view_report.cfm?srm=8012

[B206] Nie S. Emory S. R. (1997). Probing single molecules and single nanoparticles by surface-enhanced Raman scattering. Science 275, 1102–1106. 10.1126/science.275.5303.1102 9027306

[B207] Orts-Gil G. Natte K. Österle W. (2013). Multi-parametric reference nanomaterials for toxicology: state of the art, future challenges and potential candidates. RSC Adv. 3, 18202–18215. 10.1039/C3RA42112K

[B208] Pan B. Xing B. (2012). Applications and implications of manufactured nanoparticles in soils: a review. Eur. J. Soil Sci. 63, 437–456. 10.1111/j.1365-2389.2012.01475.x

[B209] Parisi C. Vigani M. Rodríguez-Cerezo E. (2015). Agricultural nanotechnologies: what are the current possibilities?. Nano Today 10, 124–127. 10.1016/j.nantod.2014.09.009

[B210] Park H. J. Kim S. H. Kim H. J. Choi S. H. (2006). A new composition of nanosized silica-silver for control of various plant diseases. Plant Pathol. J. 22, 295–302. 10.5423/PPJ.2006.22.3.295

[B211] Park J. Cha S. Cho S. Park Y. (2016). Green synthesis of gold and silver nanoparticles using gallic acid: catalytic activity and conversion yield toward the 4-nitrophenol reduction reaction. J. Nanopart. Res. 18, 166 10.1007/s11051-016-3466-2

[B212] Park K. Tuttle G. Sinche F. Harper S. L. (2013). Stability of citrate-capped silver nanoparticles in exposure media and their effects on the development of embryonic zebrafish (*Danio rerio*). Arch. Pharm. Res. 36, 125–133. 10.1007/s12272-013-0005-x 23325492 PMC4029426

[B213] Pastoriza-Santos I. Liz-Marzán L. M. (1999). Formation and stabilization of silver nanoparticles through reduction by N,N-Dimethylformamide. Langmuir 15, 948–951. 10.1021/la980984u

[B214] Patenaude J. Legault G. A. Beauvais J. Bernier L. Be'land J. P. Boissy P.. (2015). Framework for the Analysis of nanotechnologies'impacts and ethical acceptability: basis of an interdisciplinary approach to assessing novel technologies. Sci. Eng. Ethics 21, 293–315. 10.1007/s11948-014-9543-y 24728612 PMC4371817

[B215] Patri A. Umbreit T. Zheng J. Nagashima K. Goering P. Francke-Carroll S.. (2009). Energy dispersive X-ray analysis of titanium dioxide nanoparticle distribution after intravenous and subcutaneous injection in mice. J. Appl. Toxicol., 29, 662–672. 10.1002/jat.1454 19626582

[B216] Pease L. F. III. Tsai D. H. Zangmeister R. A. Zachariah M. R. Tarlov M. J. NIST–NCL Method PCC-5 (2010). Analysis of Gold Nanoparticles by Electrospray Differential Mobility Analysis. Gaithersburg, MD: National Institute of Standards and Technology. 39012982

[B217] Pecher J. Mecking S. (2010). Nanoparticles of Conjugated Polymers. Chem. Rev. 110, 6260–6279. 10.1021/cr100132y 20684570

[B218] Phogat N. Ali Khan S. Shankar S. Ansary A. A. Uddin I. (2016). Fate of inorganic nanoparticles in agriculture. Adv. Mater. Lett. 7, 03–12. 10.5185/amlett.2016.6048

[B219] Piccinno F. Gottschalk F. Seeger S. Nowack B. J. (2012). Industrial production quantities and uses of ten engineered nanomaterials in Europe and the world. J Nanopart. Res. 14, 1109 10.1007/s11051-012-1109-9

[B220] Picotto G. B. Koenders L. Wilkening G. (2009). Nanoscale metrology. Meas. Sci. Technol. 20:080101 10.1088/0957-0233/20/8/080101

[B221] Pinto V. Ferreira M. J. Silva R. Santos H. A. Silva S. F. Pereira C. M. (2010). Long time effect on the stability of silver nanoparticles in aqueous medium: effect of the synthesis and storage conditions. Colloids Surf. A 364, 19–25. 10.1016/j.colsurfa.2010.04.015

[B222] Pulit-Prociak J. Banach M. (2016). Silver nanoparticles–a material of the future…?. Open Chem. 14, 76–91. 10.1515/chem-2016-0005

[B223] Pyatenko A. Yamaguchi M. Suzuki M. (2007). Synthesis of spherical silver nanoparticles with controllable sizes in aqueous solutions. J. Phys. Chem. C 111, 7910–7917. 10.1021/jp071080x

[B224] Pyrz W. D. Buttrey D. J. (2008). Particle size determination using TEM: a discussion of image acquisition and analysis for the novice microscopist. Langmuir 24, 11350–11360. 10.1021/la801367j 18729338

[B225] Qu Y. Ma Y. (2012). A simple approach towards uniform spherical Ag-like nanoparticles. Nanoscale 4, 3036–3039. 10.1039/C2NR30532A 22508561

[B226] Quadros M. E. Pierson I. V. R. Tulve N. S. Willis R. Rogers K. Thomas T. A.. (2013). Release of silver from nanotechnology-based consumer products for children. Environ. Sci. Technol. 47, 8894–8901. 10.1021/es4015844 23822900

[B227] Rasmussen K. Mast J. De Temmerman P. J. Verleysen E. Waegeneers N. Van Steen F. (2014). Titanium dioxide, NM-100, NM-101, NM-102, NM-103, NM-104, NM-105: Characterisation and Physico-Chemical Properties. JRC Science and Policy Reports. EUR 26637 EN.

[B228] Reddy L. H. Arias J. L. Nicolas J. Couvreur P. (2012). Magnetic nanoparticles: design and characterization, toxicity and biocompatibility, pharmaceutical and biomedical applications. Chem. Rev. 112, 5818–5878. 10.1021/cr300068p 23043508

[B229] Regulation (EC) No 1223/2009 of the European Parliament of the Council of 30 November 2009 on cosmetic products Off. J. Eur. Union. L342, (2009) 59–209. Available online at: http://eur-lex.europa.eu/

[B230] Regulation (EU) 2015/2283 of the European Parliament of the Council of 25 November 2015 on novel foods amending Regulation (EU) No 1169/2011 of the European Parliament of the Council repealing Regulation (EC) No 258/97 of the European Parliament of the Council Commission Regulation (EC) No 1852/2001 Off. J. Eur. Union. L327, (2015) 1–22. Available online at: http://eur-lex.europa.eu/

[B231] Regulation (EU) No 528/2012 of the European Parliament of the Council of 22 May 2012 concerning the making available on the market use of biocidal products Off. J. Eur. Union. L167, (2012) 1–123. Available online at: http://eur-lex.europa.eu/

[B232] Regulation (EU) No 1363/2013 of the Commission Delegated Regulation of 12 December 2013 amending Regulation (EU) No 1169/2011 of the European Parliament of the Council on the provision of food information to consumers as regards the definition of ‘engineered nanomaterials’. No 1363/2013. Off. J. Eur. Union L. 343 26 Available online at: http://eur-lex.europa.eu/.

[B233] Rice S. B. Chan C. Brown S. C. Eschbach P. Han L. Ensor D. S.. (2013). Particle size distributions by transmission electron microscopy: an interlaboratory comparison case study. Metrologia 50:663. 10.1088/0026-1394/50/6/663 26361398 PMC4562322

[B234] Rodrıguez-Sanchez L. Blanco M. C. Lopez-Quintela M. A. (2000). Electrochemical synthesis of silver nanoparticles. J. Phys. Chem. B 104, 9683–9688. 10.1021/jp001761r

[B235] Roebben G. Kestens V. Varga Z. Charoud-Got J. Ramaye Y. Gollwitzer C.. (2015). Reference materials and representative test materials to develop nanoparticle characterization methods: the NanoChOp projectcase. Front. Chem. 3:56. 10.3389/fchem.2015.00056 26539428 PMC4609882

[B236] Roebben G. Ramirez-Garcia S. Hackley V. A. Roesslein M. Klaessig F. Kestens V. (2011). Interlaboratory comparison of size and surface charge measurements on nanoparticles prior to biological impact assessment. J. Nanopart. Res. 13, 2675–2687. 10.1007/s11051-011-0423-y

[B237] Roebben G. Rasmussen K. Kestens V. Linsinger T. P. J. Rauscher H. Emons H. (2013). Reference materials and representative test materials: the nanotechnology case. J. Nanopart. Res. 15, 1–13. 10.1007/s11051-013-1455-2

[B238] Römer I. White T. A. Baalousha M. Chipman K. Viant M. R. Lead J. R. (2011). Aggregation and dispersion of silver nanoparticles in exposure media for aquatic toxicity tests. J. Chromatogr. A 1218, 4226–4233. 10.1016/j.chroma.2011.03.034 21529813

[B239] Rosenman K. D. Moss A. Kon S. (1979). Argyria: clinical implications of exposure to silver nitrate and silver oxide. J. Occup. Med. 21, 430–435. 469606

[B240] Sargent J. F. Jr. (2016). Nanotechnology: A Policy Primer. CRS Report. Congressional Research Service.

[B241] Scanlan L. D. Reed R. B. Loguinov A. V. Antczak P. Tagmount A. Aloni S.. (2013). Silver nanowire exposure results in internalization and toxicity to *Daphnia magna*. ACS Nano 7, 10681–10694. 10.1021/nn4034103 24099093 PMC3912856

[B242] Schlich K. Klawonn T. Terytze K. Hund-Rinke K. (2013). Effects of silver nanoparticles and silver nitrate in the earthworm reproduction test. Environ. Toxicol. Chem. 32, 181–188. 10.1002/etc.2030 23059754

[B243] Schmid O. Stoeger T. (2016). Surface area is the biologically most effective dose metric for acute nanoparticle toxicity in the lung. J Aerosp. Sci. 99, 133–143. 10.1016/j.jaerosci.2015.12.006

[B244] Segev-Bar M. Haick H. (2013). Flexible sensors based on nanoparticles. ACSnano 7, 8366–8378. 10.1021/nn402728g 23998193

[B245] Shanmugaraj K. Ilanchelian M. (2016). Colorimetric determination of sulfide using chitosan-capped silver nanoparticles. Microchim. Acta 183, 1721–1728. 10.1007/s00604-016-1802-y

[B246] Sharma H. Mishra P. K. Talegaonkar S. Vaidya B. (2015). Metal nanoparticles: a theranostic nanotool against cancer. Drug Discov. Today 20, 1143–1151. 10.1016/j.drudis.2015.05.009 26007605

[B247] Shin S. W. Song I. H. Um S. H. (2015). Role of physicochemical properties in nanoparticle toxicity. Nanomaterials 5, 1351–1365. 10.3390/nano5031351 PMC5304630 28347068

[B248] Shirtcliffe N. Nickel U. Sheneider S. J. (1999). Reproducible preparation of silver sols with small particle size using borohydride reduction: for use as nuclei for preparation of larger particles. J. Colloid Interface Sci. 211, 122–129. 10.1006/jcis.1998.5980 9929443

[B249] Singh C. Friedrichs S. Levin M. Birkedal R. Jensen K. A. Pojana G. (2011). NMSeries of Representative Manufactured Nanomaterials, Zinc Oxide NM-110, NM-111, NM-112, NM-113, Characterisation and Test Item Preparation. EUR 25066 EN, ISBN 978-92-79-22215-3

[B250] Sondi I. Goia D. V. Matijević E. (2003). Preparation of highly concentrated stable dispersions of uniform silver nanoparticles. J. Colloid Interface Sci. 260, 75–81. 10.1016/S0021-9797(02)00205-9 12742036

[B251] Song N. W. Park K. M. Lee I. H. Huh H. (2009). Uncertainty estimation of nanoparticle size distribution from a finite number of data obtained by microscopic analysis. Metrologia 46, 480 10.1088/0026-1394/46/5/012

[B252] Sperling R. A. Parak W. J. (2010). Surface modification, functionalization and bioconjugation of colloidal inorganic nanoparticles. Philos. Trans. R. Soc. A 368, 1915. 10.1098/rsta.2009.0273 20156828

[B253] Stebounova L. V. Guio E. Grassian V. H. (2011). Silver nanoparticles in simulated biological media: a study of aggregation, sedimentation, and dissolution. J. Nanopart. Res. 13, 233–244. 10.1007/s11051-010-0022-3

[B254] Stefaniak A. B. Hackley V. A. Roebben G. Ehara K. Hankin S. Postek M. T.. (2013). Nanoscale reference materials for environmental, health and safety measurements: needs, gaps and opportunities. Nanotoxicology 7, 1325–1337. 10.3109/17435390.2012.739664 23061887

[B255] Steinigeweg D. Schlücker S. (2012). Monodispersity and size control in the synthesis of 20–100 nm quasi-spherical silver nanoparticles by citrate and ascorbic acid reduction in glycerol–water mixtures. Chem. Commun. 48, 8682–8684. 10.1039/c2cc33850e 22822486

[B256] Stone V. Nowack B. Baun A. van den Brink N. von der Kammer F. Dusinska M.. (2010). Nanomaterials for environmental studies: classification, reference material issues, and strategies for physico-chemical characterisation. Sci. Tot. Environ. 408, 1745–1754. 10.1016/j.scitotenv.2009.10.035 19903569

[B257] Sue Y. M. Lee J. Y. Y. Wang M. C. Lin T. K. Sung J. M. Huang J. J. (2001). Generalized argyria in two chronic hemodialysis patients. Am. J. Kidney Dis. 37, 1048–1051. 10.1016/S0272-6386(05)80023-X 11325689

[B258] Sun Y. Xia Y. (2002). Shape-controlled synthesis of gold and silver nanoparticles. Science 298, 2176–2179. 10.1126/science.1077229 12481134

[B259] Takahashi K. Kato H. Saito T. Matsuyama S. Kinugasa S. (2008). Precise measurement of the size of nanoparticles by dynamic light scattering with uncertainty analysis. Part. Part. Syst. Char. 25, 31–38. 10.1002/ppsc.200700015

[B260] Thanh N. T. K. Maclean N. Mahiddine S. (2014). Mechanisms of nucleation and growth of nanoparticles in solution. Chem. Rev. 114, 7610−7630. 10.1021/cr400544s 25003956

[B261] Tiede K. Boxall A. B. Tear S. P. Lewis J. David H. Hassellöv M. (2008). Detection and characterization of engineered nanoparticles in food and the environment. Food Addit. Contam. 25, 795–821. 10.1080/02652030802007553 18569000

[B262] Tkalec Ž. P. Drobne D. Vogel-Mikuš K. Pongrac P. Regvar M. Štrus J. (2011). Micro-PIXE study of Ag in digestive glands of a nano-Ag fed arthropod (Porcellio scaber, Isopoda, Crustacea). Nucl. Instrum. Meth. Phys. Res. 269, 2286–2291. 10.1016/j.nimb.2011.02.068

[B263] Tolaymat T. M. El Badawy A. M. Genaidy A. Scheckel K. G. Luxton T. P. Suidan M. (2010). An evidence-based environmental perspective of manufactured silver nanoparticle in syntheses and applications: a systematic review and critical appraisal of peer-reviewed scientific papers. Sci. Tot. Environ. 408, 999–1006. 10.1016/j.scitotenv.2009.11.003 19945151

[B264] Tomaszewska E. Soliwoda K. Kadziola K. Tkacz-Szczesna B. Celichowski G. Cichomski M. (2013). Detection limits of DLS and UV-Vis spectroscopy in characterization of polydisperse nanoparticles colloids. J Nanomater. 2013:313081 10.1155/2013/313081

[B265] Toshima N. Yonezawa T. (1998). Bimetallic nanoparticles-novel materials for chemical and physical applications. N. J. Chem. 22, 1179–1201. 10.1039/A805753B

[B266] Tran Q. H. Nguyen V. Q. Le A. T. (2013). Silver nanoparticles: synthesis, properties, toxicology, applications and perspectives. Adv. Nat. Sci. 4, 20 10.1088/2043-6262/4/3/033001

[B267] Turkevich J. Stevenson P. C. Hillier J. (1951). A study of the nucleation and growth processes in the synthesis of colloidal gold. Discuss. Faraday Soc. 55, 75 10.1039/D.F.9511100055

[B268] U.S. Department of Health Human Resources, (2010). 12th Report on Carcinogens,, National Toxicology, Research Program, Research Triangle Park, NC, USA.

[B269] U.S. FDA (2014). U.S. Department of Health and Human Services, Food and Drug Administration (U.S. FDA), Guidance for Industry Considering Whether an FDA-Regulated Product Involves the Application of Nanotechnology, Adm: US Food Drug (2014). Available online at: http://www.fda.gov/RegulatoryInformation/Guidances/ucm257698.htm

[B270] U.S. FDA (2015). U.S. Department of Health and Human Services, Food and Drug Administration (U.S. FDA), FDA's Approach to Regulation of Nanotechnology Products, (2015). Available online at: http://www.fda.gov/ScienceResearch/SpecialTopics/Nanotechnology/ucm301114.htm#guidance

[B271] Van Hyning D. L. Zukoski C. F. (1998). Formation mechanisms and aggregation behavior of borohydride reduced silver particles. Langmuir 14, 7034–7040. 10.1021/la980325h

[B272] Vanamudan A. Sudhakar P. P. (2016). Biopolymer capped silver nanoparticles with potential for multifaceted applications. Int. J. Biol. Macromol. 86, 262–268. 10.1016/j.ijbiomac.2016.01.056 26800899

[B273] Vance M. E. Kuiken T. Vejerano E. P. McGinnis S. P. Hochella M. F. Jr. Rejeski D.. (2015). Nanotechnology in the real world: redeveloping the nanomaterial consumer products inventory. Beilstein J. Nanotechnol. 6, 1769–1780. 10.3762/bjnano.6.181 26425429 PMC4578396

[B274] Velusamy P. Su C. H. Kumar G. V. Adhikary S. Pandian K. Gopinath S. C.. (2016). Biopolymers regulate silver nanoparticle under microwave irradiation for effective antibacterial and antibiofilm activities. PLoS ONE 11:e0157612. 10.1371/journal.pone.0157612 27304672 PMC4909208

[B275] Verleysen E. Van Doren E. Waegeneers N. De Temmerman P. J. Abi Daoud Francisco M. Mast J. (2015). TEM and SP-ICP-MS analysis of the release of silver nanoparticles from decoration of pastry. J. Agric. Food Chem. 63, 3570–3578. 10.1021/acs.jafc.5b00578 25768118

[B276] Viswanatha R. Sarma D. D. (2007). Chapter 4 Growth of Nanocrystals in Solution,, in Nanomaterials Chemistry, eds Rao C. N. R. Muller A. Cheetham A. K. Weinheim: WILEY-VCH Verlag GmbH & Co. KGaA.

[B277] Vladár A. E. Ming B. (2010). Measuring the Size of Colloidal Gold Nano-particles Using High-Resolution Scanning Electron Microscopy. Gaithersburg, MD: National Institute of Standards and Technology Available online at: https://ncl.cancer.gov/sites/default/files/protocols/NCL_Method_PCC-15.pdf 39013054

[B278] Von Goetz N. Lorenz C. Windler L. Nowack B. Heuberger M. Hungerbuhler K. (2013). Migration of Ag-and TiO2-(Nano) particles from textiles into artificial sweat under physical stress: experiments and exposure modeling. Environ. Sci. Technol. 47, 9979–9987. 10.1021/es304329w 23786648

[B279] Waalewijn-Kool P. L. Klein K. Forniés R. M. van Gestel C. A. (2014). Bioaccumulation and toxicity of silver nanoparticles and silver nitrate to the soil arthropod Folsomia candida. Ecotoxicology 23, 1629–1637. 10.1007/s10646-014-1302-y 25139028

[B280] Wadhera A. Fung M. (2005). Systemic argyria associated with ingestion of colloidal silver. Dermatol. Online J. 11, 12. Available online at: https://escholarship.org/uc/item/0832g6d3 15748553

[B281] Walczyk D. Bombelli F. B. Monopoli M. P. Lynch I. Dawson K. A. (2010). What the cell “sees” in bionanoscience. J Am. Chem. Soc. 132, 5761–5768. 10.1021/ja910675v 20356039

[B282] Wan A. T. Conyers R. A. Coombs C. J. Masterton J. P. (1991). Determination of silver in blood, urine, and tissues of volunteers and burn patients. Clin. Chem. 37, 1683–1687. 1914165

[B283] Wan Y. Guo Z. Jiang X. Fang K. Lu X. Zhang Y.. (2013). Quasi-spherical silver nanoparticles: aqueous synthesis and size control by the seed-mediated Lee–Meisel method. J. Colloid Interface Sci. 394, 263–268. 10.1016/j.jcis.2012.12.037 23332939

[B284] Warner M. G. Reed S. M. Hutchison J. E. (2000). Small, water-soluble, ligand-stabilized gold nanoparticles synthesized by interfacial ligand exchange reactions. Chem. Mater. 12, 3316–3320. 10.1021/cm0003875

[B285] Watanabe K. Menzel D. Nilius N. Freund J. H. (2006). Photochemistry on Metal Nanoparticles. Chem. Rev. 106, 4301–4320. 10.1021/cr050167g 17031988

[B286] Wen R. Hu L. Qu G. Zhou Q. Jiang G. (2016). Exposure, tissue biodistribution, and biotransformation of nanosilver. Nanoimpact 2, 18–28. 10.1016/j.impact.2016.06.001

[B287] Whiteley C. M. Dalla Valle M. Jones K. C. Sweetman A. J. (2013). Challenges in assessing release, exposure and fate of silver nanoparticles within the UK environment. Environ. Sci. Process. Impact 15, 2050–2058. 10.1039/c3em00226h 24056694

[B288] Wijnhoven S. W. Peijnenburg W. J. Herberts C. A. Hagens W. I. Oomen A. G. Heugens E. H. (2009). Nano-silver–a review of available data and knowledge gaps in human and environmental risk assessment. Nanotoxicology 3, 109–138. 10.1080/17435390902725914

[B289] Wu M. Ma B. Pan T. Chen S. Sun J. (2016). Silver-nanoparticle-colored cotton fabrics with tunable colors and durable antibacterial and self-healing superhydrophobic properties. Adv. Funct. Mater. 26, 569–576. 10.1002/adfm.201504197

[B290] Xu R. Wang D. Zhang J. Li Y. (2006). Shape-dependent catalytic activity of silver nanoparticles for the oxidation of styrene. Chem. Asian J. 1, 888–893. 10.1002/asia.200600260 17441132

[B291] Xu Z. Hu G. (2012). Simple and green synthesis of monodisperse silver nanoparticles and surface enhanced Raman scattering activity. RSC Adv. 2, 11404–11409. 10.1039/c2ra21745g

[B292] Yahyaei B. Peyvandi N. Akbari H. Arabzadeh S. Afsharnezhad S. Ajoudanifar H. (2016). Production, assessment, and impregnation of hyaluronic acid with silver nanoparticles that were produced by Streptococcus pyogenes for tissue engineering applications. Appl. Biol. Chem. 59, 227–237.

[B293] Yang E. J. Kim S. Kim J. S. Choi I. H. (2012). Inflammasome formation and IL-1β release by human blood monocytes in response to silver nanoparticles. Biomaterials 33, 6858–6867. 10.1016/j.biomaterials.2012.06.016 22770526

[B294] Yang J. Yin H. Jia J. Wei Y. (2011). Facile synthesis of high-concentration, stable aqueous dispersions of uniform silver nanoparticles using aniline as a reductant. Langmuir 27, 5047–5053. 10.1021/la200013z 21434661

[B295] Yang X. Jiang C. Hsu-Kim H. Badireddy A. R. Dykstra M. Wiesner M.. (2014). Silver nanoparticle behavior, uptake, and toxicity in Caenorhabditis elegans: effects of natural organic matter. Environ. Sci. Technol. 48, 3486–3495. 10.1021/es404444n 24568198

[B296] Yen C. W. Puig H. Tam J. O. Gómez-Márquez J. Bosch I. Hamad-Schifferli K.. (2015). Multicolored silver nanoparticles for multiplexed disease diagnostics: distinguishing dengue, yellow fever, and Ebola viruses. Lab Chip 15, 1638–1641. 10.1039/C5LC00055F 25672590 PMC4375736

[B297] Yeo M. Yoon J. (2009). Comparison of the effects of nano-silver antibacterial coatings and silver ions on zebrafish embryogenesis. Mol. Cell Toxicol. 5, 23–31. Available online at: http://www.koreascience.or.kr/article/ArticleFullRecord.jsp?cn=DDODB@_2009_v5n1_23

[B298] Zamiri R. Zakaria A. Ahangar H. A. Sadrolhosseini A. R. Mahdi M. A. (2010). Fabrication of silver nanoparticles dispersed in palm oil using laser ablation. Int. J. Mol. Sci. 11, 4764–4770. 10.3390/ijms11114764 21151470 PMC3000114

[B299] Zhai Y. Hunting E. R. Wouters M. Peijnenburg W. J. Vijver M. G. (2016). Silver nanoparticles, ions, and shape governing soil microbial functional diversity: nano shapes micro. Front. Microbiol. 7:1123. 10.3389/fmicb.2016.01123 27504108 PMC4959451

[B300] Zhang T. Wang L. Chen Q. Chen C. (2014). Cytotoxic potential of silver nanoparticles. Yonsei Med. J. 55, 283–291. 10.3349/ymj.2014.55.2.283 24532494 PMC3936614

[B301] Zhao T. Sun R. Yu S. Zhang Z. Zhou L. Huang H. (2010). Size-controlled preparation of silver nanoparticles by a modified polyol method. Colloids Surf. A 366, 197–202. 10.1016/j.colsurfa.2010.06.005

